# Investigating the phosphinic acid tripeptide mimetic DG013A as a tool compound inhibitor of the M1-aminopeptidase ERAP1

**DOI:** 10.1016/j.bmcl.2021.128050

**Published:** 2021-06-15

**Authors:** Birgit Wilding, A. Elisa Pasqua, Nicola E. A. Chessum, Olivier A. Pierrat, Tamas Hahner, Kathy Tomlin, Erald Shehu, Rosemary Burke, G. Meirion Richards, Bradleigh Whitton, Esther N. Arwert, Arjun Thapaliya, Ramya Salimraj, Rob van Montfort, Agi Skawinska, Angela Hayes, Florence Raynaud, Rajesh Chopra, Keith Jones, Gary Newton, Matthew D. Cheeseman

**Affiliations:** a Cancer Research UK Cancer Therapeutics Unit, The Institute of Cancer Research, London SW7 3RP, UK; b Division of Structural Biology, The Institute of Cancer Research, London SW7 3RP, UK

**Keywords:** ERAP1, DG013A, Chemical probe, M1-aminopeptidase, Permeability

## Abstract

ERAP1 is a zinc-dependent M1-aminopeptidase that trims lipophilic amino acids from the *N*-terminus of peptides. Owing to its importance in the processing of antigens and regulation of the adaptive immune response, dysregulation of the highly polymorphic ERAP1 has been implicated in autoimmune disease and cancer. To test this hypothesis and establish the role of ERAP1 in these disease areas, high affinity, cell permeable and selective chemical probes are essential. DG013A **1**, is a phosphinic acid tripeptide mimetic inhibitor with reported low nanomolar affinity for ERAP1. However, this chemotype is a privileged structure for binding to various metal-dependent peptidases and contains a highly charged phosphinic acid moiety, so it was unclear whether it would display the high selectivity and passive permeability required for a chemical probe. Therefore, we designed a new stereoselective route to synthesize a library of DG013A **1** analogues to determine the suitability of this compound as a cellular chemical probe to validate ERAP1 as a drug discovery target.

## Graphical abstract

**Figure:**
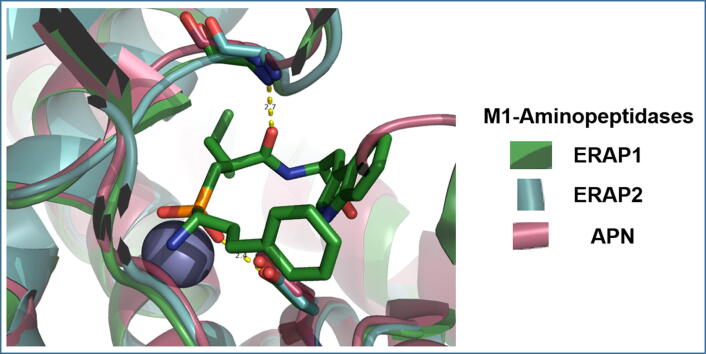

Endoplasmic reticulum aminopeptidase 1 (ERAP1) and its closely related M1-family member, ERAP2 (49% identity),^1^ are zinc-dependent hydrolase enzymes that are critical to the adaptive immune response of cells.^2^ Following the degradation of aberrant proteins by the proteasome, the resulting peptides are transported to the ER via the TAP protein complex, where they undergo further processing by ERAP1 to produce peptides of the correct length (8 to 12 amino acids) to facilitate binding to the major histocompatibility class I (MHC1) protein.^3^ This complex then translocates to the cell surface to present the peptides as antigens to be recognized by circulating *T*-lymphocytes.^4^ Dysregulation of the highly polymorphic ERAP1 has been identified as an important risk factor in disease.^5^ Several haplotypes have been demonstrated to impact the antigenic peptide repertoire, potentially resulting in autoimmune disease^6^ and possibly as a mechanism for cancer cells to evade immune detection.^7^, ^8^ Mice express an orthologue of ERAP1 with 84% sequence identity, but do not possess the ERAP2 gene.^9^ ERAP1 knockout in mouse syngeneic cancer models has resulted in tumor rejection and significantly improved survival.^10^, ^11^

Owing to its clear role in the regulation of the adaptive immune system, the pursuit of ERAP1 as a therapeutic target has received increasing attention.^12^ Because modulating the adaptive immune response is complex, well-validated, small molecule chemical probes are crucial in understanding the potential clinical setting, patient population and therapeutic index of ERAP1 inhibitors. Chemical probes are important tools in biology and drug discovery to deconvolute the role of target proteins in cellular phenotypes. However, to interpret data from chemical probes, their cellular permeability and protein selectivity profiles must be robustly established. Several chemotypes have been reported as inhibitors of ERAP1;^13^, ^14^, ^15^ however, only the phosphinic acid, transition-state mimic compounds, exemplified by the tripeptide mimetic DG013A **1** (Scheme 1), have demonstrated sub-micromolar affinity for the recombinant protein.^16^ As part of a recent study into the role of ERAP1 in cancer, we now report our investigation into the use of **1** as tool compound to study ERAP1 inhibition.

**Scheme 1:**
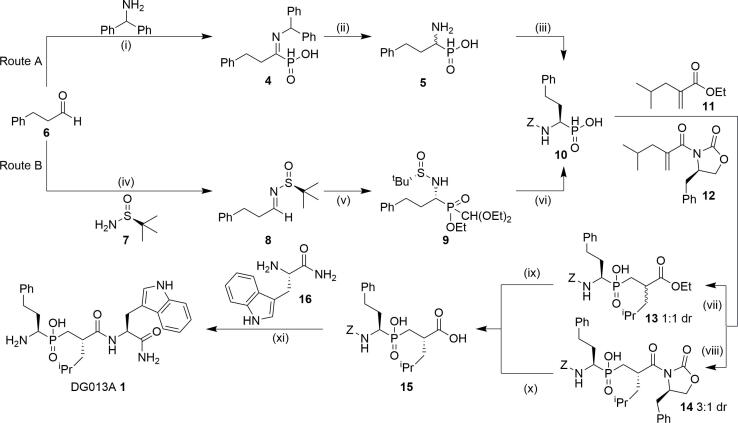
Stereoselective synthesis of the proposed ERAP1 chemical probe DG013A 1. Reagents and conditions: i) H_3_PO_2_, EtOH, 80 °C, 3 h, 68%; ii) HBr (48%), 100 °C, 3 h, 72%, iii) a) Cbz-OSu, Na_2_CO_3_, THF/H_2_O (1:1), 20 °C, 24 h, 100%; b) (*S*)-(-)-α-methylbenzylamine, EtOH, multiple recrystallizations 19%, c) 4M HCl aq. 20 °C, 3 h, 94%, iv) CuSO_4_, CH_2_Cl_2_, 20 °C, 87%; v) Rb_2_CO_3_, ethyl (diethoxymethyl)phosphinate, CH_2_Cl_2_, 20 °C, 56%; vi) a) 4M HCl aq., reflux, 93%; b) Cbz-OSu, Na_2_CO_3_, THF/H_2_O, 94%; c) *S*-(-)-α-methylbenzylamine, EtOH, multiple recrystallizations; d) 4M HCl aq., 20 °C, 33% (over two steps), vii) BSA, **11**, 20 °C, 16 h; ix) a) NaOH, EtOH, H_2_O, 20 °C, 24 h, 80% (over 2 steps); xi) a) 33% HBr in AcOH, 88%;b) Boc_2_O, Et_3_N, DMF, 78%; xi) c) EDC.HCl, HOBt, DIPEA, CH_2_Cl_2_, 20 °C, 80%; d) TFA/CH_2_Cl_2_/TIS/H_2_O, quant.; e) Separation by HPLC, **1** 31%, DG013B (**2**) 24%,  viii) a) **12**, CH_2_Cl_2_, DIPEA, TMSCl, 0 °C to RT, 20 h; b) Separation by HPLC, **14** 38%; x) H_2_O_2_, LiOH aq., 0 °C, 87%.

DG013A **1** (Table 1, Entry 1) is a pseudotripeptide phosphinic acid containing 3 stereogenic centers (((*S*)-2-(((*S*)-1-amino-3-(1*H*-indol-3-yl)-1-oxopropan-2-yl)carbamoyl)-4-methylpentyl)((*R*)-1-amino-3-phenylpropyl)phosphinic acid, the *S,S,R*-diastereomer), where only the tryptophan moiety can be obtained without asymmetric synthesis or chiral resolution. The *R,S,R*-diastereomer, which is epimeric at the central leucine-mimetic position, is described as DG013B **2** (Table 1, Entry 2) and has been used as a weakly binding negative control.^16^ Several recent studies have highlighted the importance for ERAP1 affinity of the homophenylalanine, leucine and tryptophan mimetic groups in their interactions with the S1-, S1′- and S2′-pockets, respectively.^17^, ^18^ Despite the high biochemical affinity of this chemotype reported for both ERAP1 and ERAP2,^16^ the predicted physicochemical properties are inconsistent with high cellular passive permeability (Table 1, Entry 1).^19^, ^20^ Consequently, it is unclear whether DG013A **1** can be utilized as a chemical probe to investigate the phenotype of ERAP1 inhibition in cancer cells and immunopeptidomic studies.^21^ Analysis of a recently published crystal structure of **1** bound to ERAP1 in the closed conformation (Fig. 1, PDB: 6M8P),^22^ suggests that although the ligand is entirely encapsulated by the protein, the tryptophan moiety-primary amide points towards the large solvent cavity above the active site and forms no direct interactions. Therefore, we hypothesized that changes to this region could potentially modulate the physicochemical properties of the ligand; whilst maintaining the interactions in the peptide residue pockets and the polar contacts within the terminal amine pocket, the zinc ion, the catalytic tyrosine-438 and glycine-317 in the GAMEN loop. Because of the perceived importance of the stereochemical purity to the binding of **1** to ERAP1, and our desire to make analogues at the tryptophan primary amide, we re-examined the published synthetic route for this complex and challenging target.

**Table 1:** Library of phosphinic acid peptidomimetics.

Entry	Compd	Structure	ERAP1^a^	ERAP2^b^	APN^c^	CACO2^e^		Physicochemical^h^	
			pIC_50_ ± SEM IC_50_ (n)^d^			P_app_ A:B (×10^-6^ cm/s)^f^	ER^g^	LogD_7.4_	*pK_a_*
1	DG013A **1**		6.73 ± 0.04 0.19 µM (17)	5.53 ± 0.03 3.0 µM (15)	8.44 ± 0.07 0.0037 µM (3)	<1.0	NA	1.0	3.6
2	DG013B **2**		4.76 ± 0.06 18 µM (5)	4.01 ± 0.09 98 µM (3)	6.65 ± 0.03 0.23 µM (3)	<1.0	NA	1.0	3.6
3	**3**		4.11 ± 0.03 77 µM (3)	<3.66 >220 µM (3)	5.47 ± 0.05 3.4 µM (3)	<1.0	>12	3.2	5.4
4	**17**		6.85 ± 0.07 0.14 µM (3)	6.05 ± 0.11 0.90 µM (3)	8.81 ± 0.01 0.0015 µM (3)	<1.0	NA	1.9	3.6
5	**18**		6.38 ± 0.09 0.41 µM (3)	5.63 ± 0.12 2.4 µM (3)	8.64 ± 0.11 0.0023 µM (3)	<1.0	NA	−1.4	3.3
6	**19**		6.49 ± 0.11 0.33 µM (3)	5.90 ± 0.07 1.3 µM (4)	8.06 ± 0.07 0.0086 µM (3)	1.5	8	2.2	3.6

See SI for details. NA = not applicable. All assay data were reprocessed using GraphPad Prism 7.04 in duplicate and fit using a four parameter variable slope non-linear regression model to estimate the IC_50_ under initial rate conditions. ERAP1 and ERAP2 proteins were produced in house, APN was purchased from Sino Biologics. ^a^ERAP1 (5 nM) was assayed using L-AMC (250 µM), the K_m_ of L-AMC was estimated to be >400 µM. ^b^ERAP2 (10 nM) was assayed using R-AMC (10 µM), the K_m_ of R-AMC was estimated to be 10 µM. ^c^APN (0.96 nM) was assayed using A-AMC (100 µM), the K_m_ of A-AMC was assumed to be 100 µM. ^d^pIC_50_ = −log IC_50_ (M) quoted to 2 dp, SEM = standard error of the mean, n = number of independent biological repeats, the IC_50_ is the geometric mean quoted to 2 sf. ^e^Caco-2 data was produced in house using standard methods, the limit of quantification P_app_ = 1.0 x 10^−6^ cm/s, all values are quoted to 2 sf. ^f^P_app_ was calculated under sink conditions using standard methods, A:B = apical to basolateral. ^g^ER = efflux ratio, calculated from P_app_B:A/P_app_A:B, an ER could not be determined when P_app_B:A<1.0x10^−6^ cm/s. ^h^LogD_7.4_ and pK_a_ were calculated using Moka 3.1.3, the pK_a_ describes the phosphinic acid only, all values are quoted to 2 sf.

**Fig. 1:**
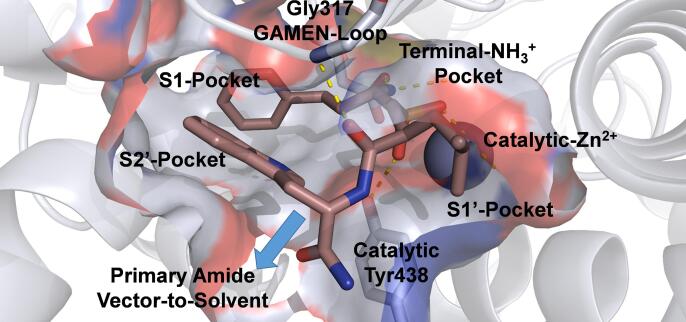
Crystal Structure of DG013A 1 bound to ERAP1 in the closed conformation. (PDB: 6M8P) – the primary amine binds in the N-terminal pocket; the phosphinic acid binds the zinc and interacts with the catalytic tyrosine-438; the backbone amide binds the GAMEN-loop; the homo-phenylalanine, leucine and tryptophan mimetics bind in their respective pockets; the primary amide motif points towards solvent and forms no direct interactions with the protein. Picture was produced using The PyMOL Molecular Graphics System, Version 2.0 Schrödinger, LLC. Red = Oxygen, Blue = Nitrogen, Orange = Phosphorus; hydrogens and solvent have been omitted for clarity.

The synthesis of DG013A **1** was first described by Stratikos et al. in 2013,^16^ although very similar analogues to Cbz-protected primary amine **3** (Table 1, Entry 3), were reported as inhibitors of matrix metalloproteinases in 1999.^23^ To obtain stereochemically pure **1**, we first focused on the homophenylalanine transition state mimic phosphinic acid precursor **10**. Consistent with previous reported syntheses (Scheme 1, Route A),^16^, ^18^, ^23^, ^24^ we generated intermediate **10** by phosphine addition to the prochiral imine precursor **4**. Following hydrolysis and re-protection of the primary amine as a Cbz-group, the racemic phosphinic acid (*rac*)-**10** was subjected to kinetic resolution via recrystallization from ethanol using *S*-(-)-α-methylbenzylamine, to generate a diastereomeric salt as had been previously described.^18^ The enantiopurity of this compound had only been determined using optical rotation (literature [α]_D_^20^ = −35, c = 1, EtOH),^18^ which was not suitable to accurately assess the enantiomeric ratio (er). Therefore, we developed a chiral high performance liquid chromatography (HPLC) method to measure the er (see SI for details).^25^ Following multiple recrystallizations, a stable optical rotation consistent with the literature value was obtained, but our chiral HPLC analysis demonstrated this kinetic resolution methodology could only result in an er = 5.8:1 for *R*-**10**, consistent with the observation by Mucha et al. that *enantio*-enrichment using recrystallization can be challenging with this intermediate.25b

To improve the enantiomeric purity of the critical precursor *R*-**10**, we developed an enantioselective route using Ellman’s chiral sulfinamide (Scheme 1, Route B). Condensation between 3-phenylpropanal **6** and (*S*)-2-methylpropane-2-sulfinamide **7** gave imine **8**, which was then reacted with (diethoxymethyl)phosphinate, using Rb_2_CO_3_ as a base, to afford diastereomeric phosphinate **9**. Following acidic hydrolysis and subsequent protection of the primary amine as a Cbz-group, the chiral phosphinic acid *R*-**10** was obtained in high er (up to 12.9:1, 150 mg scale) as determined by chiral HPLC; although the er obtained for this transformation was typically dependent on the scale the reaction was performed, with the er decreasing with larger scale synthesis (8.4:1, 2 g scale, see SI for details). Recrystallization from ethanol and (*S*)-(-)-α-methylbenzylamine using this enantioenriched material now gave the product *R*-**10** as a single observable enantiomer (>99:1 er).

Synthesis of the second chiral center was carried out via a phospha-Michael addition to acrylate **11**. Following a small screen of reaction conditions, we discovered that using *N,O*-bis(trimethylsilyl)acetamide as the reaction solvent gave complete and clean conversion to the required product in high yield but only in a 1:1 dr, despite the potential for diastereoselectivity in this transformation. Alternatively, the phospha-Michael addition could be carried out in a diastereomerically enriched manner using (*R*)-4-benzyloxazolidin-2-one derived **12** as a chiral auxiliary to give, following hydrolysis, the desired *S,R*-epimer acid **15** in a 3:1 dr, although on scale completing the non-stereoselective synthesis was more efficient. Importantly, the epimeric mixtures at the leucine mimetic position could be readily separated by chromatography; however, the epimers at the homophenylalanine position could not, highlighting the importance of the enantioselective synthesis of *R*-**10** in the final stereochemical purity of **1**.^26^

The chiral acid **15** was then reacted with the primary amide derivative of tryptophan **16** under standard coupling conditions. Finally, deprotection and purification using a modified previously described procedure,^18^ gave DG013A **1** in high enantiomeric and diastereomeric purity (>99:1).^27^ Using these methods, we synthesized a small library of DG013A **1** analogues, varying the tryptophan primary amide substituent (Table 1).

We utilized standard X-AMC assays (see SI for details) to characterize the binding of our peptidomimetic phosphinic acid library to the M1-aminopeptidases.^28^ Conditions were selected whereby substrate concentrations were at or below K_m_, so that IC_50_ values could be compared between proteins for competitive ligands. DG013B **2** (Table 1, Entry 2) and the Cbz-protected analogue **3** (Table 1, Entry 3), which blocks binding to the primary amine pocket, displayed very weak affinity for ERAP1 and ERAP2, consistent with previously described studies.^16^ However, in our hands, DG013A **1** (Table 1, Entry 1) displayed significantly weaker affinity for both ERAP1 (lit. IC_50_ = 33 nM)^16^ and ERAP2 (lit. IC_50_ = 11 nM)^16^ than had been previously reported, with > 5-fold increase in IC_50_. l-AMC displays very weak affinity for ERAP1, which makes estimating its K_m_, and therefore the IC_50_ shift due to substrate competition, difficult to determine. However, the concentration of l-AMC and R-AMC used in our assays were similar to reported values.^28^ Possibly, the highly polymorphic nature of the ERAP proteins and the large conformational flexibility of the binding site, could make measured IC_50_s highly haplotype and condition dependent, and therefore direct comparisons difficult. Also, our assay was run at pH8 and it is possible that the biochemical activity of phosphinic acid ERAP inhibitors is pH-dependent, owing to changes in the conformational preference. Nonetheless, **1** did display moderate potency against ERAP1, so it could still have potential utility as a chemical tool in cellular assays to study the role of ERAP1 inhibition in antigen presentation and immunopeptidomics.^21^

Whether the inhibition of ERAP1 by **1** would translate to in-cell activity would depend on its permeability, as well as its affinity. Therefore, compound **1** was analyzed in the commonly used Caco-2 assay, where we found its passive permeability was negligible and below our limit of quantification (P_app_ < 1.0×10^−6^ cm/s). The low passive permeability of **1** is consistent with the predicted physicochemical properties of this chemotype, with a low calculated LogD_7.4_ and highly acidic phosphinic acid moiety (Moka *pK_a_* = 3.6).^29^

To improve the physicochemical properties of this series, we investigated the solvent exposed primary amide motif. Hydrolysis of the amide to the carboxylic acid gave **18** (Table 1, Entry 5), which resulted in only a modest 2-fold drop in ERAP1 activity compared to **1**, but unsurprisingly, no improvement in passive permeability. The methyl ester **17** (Table 1, Entry 4) regained the ERAP1 activity but again displayed no measurable improvement in passive permeability. Therefore, we removed the amide moiety to give **19** (Table 1, Entry 6), which displayed similar ERAP1 potency to **1**, consistent with our structure-based design. However, with its increased lipophilicity and loss of the highly solvated amide moiety, **19** possessed measurable, although still very low, passive permeability in the Caco-2 assay.

With its increased permeability, whether **19** is a useful probe to investigate ERAP1 inhibition in cells would finally depend on its selectivity.^30^, ^31^ All members of the M1-aminopeptidase family possess highly conserved structural similarity.^32^ To investigate the selectivity of **19** we selected aminopeptidase N (APN, 31% identity)^33^ as a representative member for our counter-screen. APN is a well-studied protein and drug discovery target.^34^ The inhibition of APN is known to lead to cytotoxicity, so it would be an undesirable target for an ERAP1 probe. APN was shown to be potently inhibited by the phosphinic acid chemotype using an A-AMC assay (see SI for details);^35^ **19** displayed an IC_50_ = 8.6 nM, 40-fold lower than both ERAP1 and ERAP2. The high affinity for APN was reflected across our phosphinic acid library, including for the proposed ERAP1 intracellular probe DG013A **1**, with an IC_50_ = 3.7 nM, 62-fold more potent than versus ERAP1. Weak antiproliferative activity was observed in HCT116 cells when treated with **1**, which could be indicative of off-target toxicity limited by its low permeability (see SI). Comparing the **1**/ERAP1 structure to overlays with ERAP2 (PDB: 4JBS) and APN (PDB: 4FYR),^36^ confirmed high structural and shape similarity, despite only moderate sequence identity (Fig. 2 and SI). Within the active site of each protein, residues that form the S1-, S1′- and S2′-pockets share little similarity; however, the key polar interactions, such as the catalytic tyrosine, are highly conserved, which is consistent with the observed polypharmacology.

**Fig. 2:**
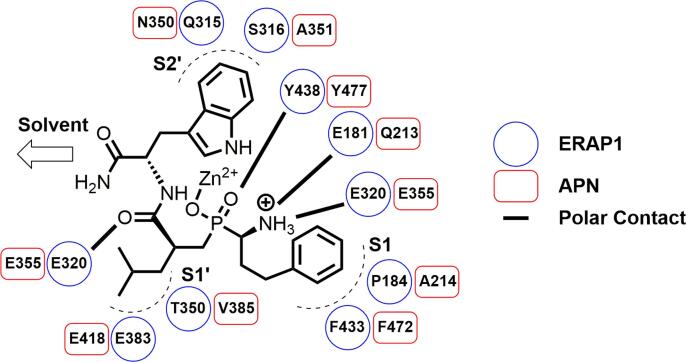
Comparison of key residues in the binding of DG013A 1 to ERAP1 and APN. Adapted from an overlay of PDB: 6M8P and 4FYR using Pymol.

In conclusion, DG013A **1** is a proposed potent inhibitor of the M1-aminopeptidases, ERAP1 and ERAP2, and has been used as a chemical probe to investigate the role of these proteins in the modulation of the antigenic peptide repertoire on cell surfaces. We developed a new stereoselective synthetic route to the crucial phosphinic acid intermediate and obtained **1** as a single epimer. Analysis of the cellular passive permeability of **1** indicated that it was negligible, which is inconsistent with translatable intracellular ERAP1 inhibition and with its use as a chemical tool. Broad ERAP1 structure activity relationships (SAR) were confirmed at the amide vector and led to **19**, an analogue with measurable, but very low, cellular passive permeability and comparable potency to **1**. However, screening **19** against the closely structurally related oncology target M1-aminopetidase, APN, revealed this chemotype was a highly potent inhibitor, a result that was repeated with all members of our phosphinic acid library. The potential of this chemotype as a chemical probe in cells is limited, although it does retain utility in biochemical assays and as a crystallography tool. DG013A **1** will likely require very long cellular exposures and will suffer from competing off-target activities, such that any effect on antigen presentation attributed to ERAP1 inhibition through using these compounds should be treated with caution. The validation of ERAP1 as a drug discovery target in autoimmune disease and cancer still requires the development of potent, permeable and selective chemical tools.

## Author contributions

N.E.A.C., B.W. and A.E.P. synthesized the compounds; O.A.P., T.H, K.T. E.S. and R.B. designed and carried out the biochemical assays; G.M.R. developed the HPLC methods and measured physicochemical properties of analogues; A.T., R.S. and R.V.M. synthesized proteins and analyzed crystal structures; B. Wh. carried out the proliferation assays; A.S., A.H. and F.R. carried out the permeability assays; N.E.A.C., B.W., A.E.P., E.A, R.C., K.J., G.N. and M.D.C. carried out data analysis and ligand design; N.E.A.C., B.W. and M.D.C. wrote the manuscript.

## Declaration of Competing Interest

The authors declare the following financial interests/personal relationships which may be considered as potential competing interests: The authors are employees of ICR and ICR has a commercial interest in inhibitors of ERAP1. The ICR operates a Rewards to Discoverers scheme, which provides financial rewards to inventors in the event of commercial licensing.
