# Supplementary material for: Investigating the phosphinic acid tripeptide mimetic DG013A as a tool compound inhibitor of the M1-aminopeptidase ERAP1
Source: Bioorg Med Chem Lett. 2021 Jun 15;42:128050. doi: 10.1016/j.bmcl.2021.128050 (PMC8188423; doi:10.1016/j.bmcl.2021.128050)
Supplement: Supplementary data 2 [file mmc2.docx]

**Supporting Information**

**Investigating the phosphinic acid tripeptide mimetic DG013A as a tool compound inhibitor of the M1-aminopeptidase ERAP1**

Birgit Wilding, A. Elisa Pasqua, Nicola E. A. Chessum, Olivier A. Pierrat, Tamas Hahner, Kathy Tomlin, Erald Shehu, Rosemary Burke, G. Meirion Richards, Bradleigh Whitton, Esther N. Arwert, Arjun Thapaliya, Ramya Salimraj, Rob van Montfort, Agnieszka Skawińska, Angela Hayes, Florence Raynaud, Rajesh Chopra, Keith Jones, Gary Newton, Matthew D. Cheeseman.

**Table of Contents**

General Experimental 2-3

Chemistry Experimental 4-21

NMR Spectra and LCMS traces of Final Compounds 22-33

M1-Aminopeptidase Crystal Structures 34

Biochemical Experimental 35-37

Permeability Assay 38

Proliferation Assay 39

Calculated Physicochemical Properties 40

References 41

**General Experimental**

**Column chromatography** was performed on a Biotage SP1 or Biotage Isolera Four purification system using Biotage Flash silica cartridges (SNAP KP-Sil) for normal phase purifications or SNAP Ultra C18 cartridges for reversed-phase purifications.

**Semi-preparative HPLC:** 500 µL or 1 mL standard injections (with needle wash) of the sample were made onto an ACE 5 C18-PFP column (5 µm, 250 x 10 mm, 250 x 21.2 mm or 250 x 30 mm Advanced Chromatography Technologies, Aberdeen, UK). Chromatographic separation at room temperature was carried out using a 1200 Series Preparative HPLC (Agilent, Santa Clara, USA). Gradient I (Grad15min20mls.m, Grad15min40mls.m): 15 minute gradient elution from 90:10 to 0:100 water:methanol (both modified with 0.1% formic acid) at a flow rate of 20 or 40 mL/min. Gradient II (Grad15min20mlsLipo.m, Grad15min40mlsLipo.m): 15 minute gradient elution from 60:40 to 0:100 water:methanol (both modified with 0.1% formic acid) at a flow rate of 20 or 40 mL/min. Gradient III: 20 minute gradient elution, 55% to 35% solvent A in solvent B in 15 minutes, then decrease to 0% solvent A in 4 minutes, and return to 55% solvent A in 0.1 minute, solvent A is 90% water with 0.1% TFA plus 10% acetonitrile, solvent B is 10% water with 0.09% TFA plus 90% acetonitrile, flow 5 mL/min. UV-Vis spectra were acquired at 230 nm on a 1200 Series Prep Scale diode array detector (Agilent, Santa Clara, USA). Post-UV and pre-MS splitting was achieved using an Active Split (Agilent, Santa Clara, USA) before being infused into a 6120 Series Quad mass spectrometer fitted with an ESI/APCI Multimode ionization source (Agilent, Santa Clara, USA). LC eluent and nebulizing gas was introduced into the grounded nebulizer with spray direction orthogonal to the capillary axis. 2 kV was applied to the charging electrode to generate a charged aerosol. The aerosol was dried by infrared emitters (200 °C) and heated drying gas (12 L/min of nitrogen at 350 °C, 60 psi), producing ions by ESI. Aerosol and ions were transferred by nebulizing gas to the APCI zone where infrared emitters vaporized solvent and analyte. A corona discharge was produced between the corona needle and APCI counter electrode by applying a current of 4 µA, ionizing the solvent to transfer charge to analyte molecules, producing ions by APCI. ESI and APCI ions simultaneously entered the transfer capillary along which a potential difference of 4 kV was applied. The fragmentor voltage was set at 175 V and skimmer at 65 V. Signal was optimized by AutoTune.m. Profile mass spectrometry data was acquired in positive ionization mode over a scan range of m/z 60-1000 (scan rate 1.0). Collection was triggered by UV signal and collected on a 1200 Series Fraction Collector (Agilent, Santa Clara, USA). Raw data was processed using Agilent Chemstation Software B.02.01.

**NMR spectra:** ^1^H-NMR spectra were recorded on Bruker Avance 500 (500 MHz) or Bruker Avance NEO 600 spectrometers (600 MHz) using an internal deuterium lock. Chemical shifts are quoted in parts per million (ppm) using the following internal references: CDCl_3_ (δH 7.26), CD_3_OD (δH 3.31) and DMSO-d_6_ (δH 2.50). Signal multiplicities are recorded as singlet (s), doublet (d), triplet (t), quartet (q) and multiplet (m), doublet of doublets (dd), doublet of doublet of doublets (ddd), broad (br) or obscured (obs). Coupling constants, *J*, are measured to the nearest 0.1 Hz. ^13^C-NMR spectra were recorded on Bruker Avance 500 (126 MHz) or Bruker Avance NEO 600 (151 MHz) spectrometers using an internal deuterium lock. Chemical shifts are quoted to 0.01 ppm, unless greater accuracy was required, using the following internal references: CDCl_3_ (δC 77.0), CD_3_OD (δC 49.0) and DMSO-d_6_ (δC 39.5). ^31^P NMR spectra were recorded on Bruker Avance 500 spectrometers at 202 MHz. ^31^P chemical shift was calibrated using Bruker instrument standard automatic calibration, which is based on deuterated lock signals and Universal referencing as outlined by IUPAC guideline (2001).

**HRMS analysis** (high resolution mass spectra) was performed on an Agilent 1200 series HPLC and diode array detector coupled to a 6210 time of flight mass spectrometer with dual multimode APCI/ESI source. Analytical separation was carried out at 30°C on a Merck Chromolith Flash column (RP-18e, 25 x 2 mm) using a flow rate of 0.75 mL/min in a 4 minute gradient elution with detection at 254 nm. The mobile phase was a mixture of methanol (solvent A) and water (solvent B), both containing formic acid at 0.1%. Gradient elution was as follows: 5:95 (A/B) to 100:0 (A/B) over 2.5 min, 100:0 (A/B) for 1 min, and then reversion back to 5:95 (A/B) over 0.1 min, finally 5:95 (A/B) for 0.4 min. HRMS references: caffeine [M+H]^+^ 195.087652; hexakis (2,2-difluroethoxy)phosphazene [M+H]^+^ 622.02896; and hexakis(1*H*,1*H*,3*H*-tetrafluoropentoxy)phosphazene [M+H]^+^ 922.009798.

**Chiral HPLC (Method A):** The chiral HPLC method to separate precursor A ((*RS*)-**5**) was adapted from the method reported by Lӓmmerhofer et al.^[[1]](#endnote-1)^ Column: Chiralpak QD-AX, 150 x 4.6 mm, solvents, MeOH/AcOH/NH_4_OAc = 98/2/0.5 (v/v/w), isocratic, flow rate 1 mL/min, sample concentration: 1 mg/mL in methanol.

**HPLC (Method B): (Separation of DG013A and DG013B):** The preparative HPLC method used an isocratic gradient. Column: ACE 5 C18-PFP column, 250 x 4.6 mm, 20% solvent A in solvent B (where solvent A = 10% MeCN in 90% H_2_O + 0.1% formic acid, solvent B = 10% H_2_O in 90% MeCN + 0.1% formic acid). To afford good separation, it was necessary to dissolve the crude product in 75:25 solvent A:B for injection onto the column and to load the compound batch-wise (5 mg aliquots).

**HPLC (Method C): (Separation of oxazolidinones 14 and S11):** Column: ACE 5 C18-PFP column 250 x 30 mm, 70-90% gradient over 15 mins of solvent A in solvent B. Where solvent A = H_2_O + 0.1% formic acid and solvent B = MeOH + 0.1% formic acid, flow rate 30 mL/min.

**Chemistry Experimental**

Scheme 1: Route A, Preparation of **10**

a) H_3_PO_2_, EtOH, 80 ^o^C, 3 h, 68%; b) HBr (48%), 100 ^o^C, 3 h, 72%, c) Cbz-OSu, Na_2_CO_3_, THF/H_2_O (1:1), 20 ^o^C, 24 h, 100%; d) (*S*)-(-)-α-methylbenzylamine, EtOH, multiple recrystallizations 19%, e) 4M HCl aq. 20 ^o^C, 3 h, 94%

((1-benzhydrylamino)-3-phenylpropyl)phosphinic acid **4**

((1-benzhydrylamino)-3-phenylpropyl)phosphinic acid **4** was prepared based on the method described by Baylis et al. ^[[2]](#endnote-2)^ Phosphinic acid (1.61 mL, 7.59 mmol) was diluted with ethanol (2 mL) and diphenylmethanamine (benzhydrylamine, 1.31 mL, 7.59 mmol) was added. The resulting reaction mixture was stirred at room temperature for 10 minutes. 3-phenylpropanal (hydrocinnamaldehyde, 1.0 mL, 7.59 mmol) was added and the resulting clear, colorless solution was heated to reflux. Upon heating, the reaction mixture turned yellow and a large amount of white precipitate formed. Within 10 minutes the reaction mixture had solidified. Additional ethanol (4 mL) was added to allow stirring. The reaction mixture was refluxed for 3 hours. Then the reaction mixture was cooled to 0 ºC. The product was isolated by filtration and washed with ethanol and diethyl ether (white solid, 1.87 g, 68%). HRMS (ESI^+^): calcd for C_22_H_24_NNaO_2_P (M + Na)^+^ 388.1437, found 388.1426. ^1^H NMR (500 MHz, DMSO-d_6_) δ 7.46 (dd, *J* = 19.3, 7.2 Hz, 4H), 7.33 (q, *J* = 7.8 Hz, 4H), 7.28 – 7.18 (m, 4H), 7.14 (t, *J* = 7.3 Hz, 1H), 7.07 (d, *J* = 7.1 Hz, 2H), 6.93 (d, *J* = ~510 Hz, 1H), 5.49 (s, 1H), 2.75 (ddd, *J* = 14.5, 9.5, 6.3 Hz, 1H), 2.61 (ddd, *J* = 14.0, 9.6, 6.1 Hz, 1H), 1.99 – 1.77 (m, 2H), one proton obscured by DMSO peak.

((*RS*)-1-amino-3-phenylpropyl)phosphinic acid **5**

(1-amino-3-phenylpropyl)phosphinic acid **5** was prepared based on the method reported by Baylis et al.^ii^ HBr (48% in water, 10 mL) was added to (1-benzhydrylamino)-3-phenylpropyl)phosphinic acid **4** (1.34 g, 3.65 mmol) and the resulting reaction mixture was heated to 100ºC for two hours. After approximately 15 minutes, all solids had dissolved and two layers were visible. After two hours the reaction mixture was concentrated. The remaining residue was partitioned between water and diethyl ether. The layers were separated and the aqueous layer was washed with diethyl ether (x 2). The aqueous layer was then concentrated to dryness. The remaining residue was diluted with 15 mL ethanol. Then, propylene oxide was added dropwise to the stirred solution until a white precipitate formed. The reaction mixture was stirred at room temperature overnight to allow complete precipitation. The product was isolated by filtration and washed with diethyl ether (white solid, 526 mg, 72%). HRMS (ESI^+^): calcd for C_9_H_15_NO_2_P (M + H)^+^ 200.0835, found 200.0840. ^1^H NMR (500 MHz, D_2_O + Na_2_CO_3_) δ 7.43 – 7.25 (m, 5H), 7.24 – 6.15 (m, 1H), 2.93 – 2.76 (m, 1H), 2.76 – 2.64 (m, 1H), 2.64 – 2.50 (m, 1H), 1.97 (dt, *J* = 16.3, 9.1 Hz, 1H), 1.67 (td, *J* = 14.7, 7.4 Hz, 1H). ^13^C NMR (126 MHz, D_2_O + Na_2_CO_3_) δ 167.41 (Na_2_CO_3_), 142.15, 128.69, 128.55, 126.12, 51.37 (d, *J* = 103.7 Hz), 50.17 (d, *J* = 99.1 Hz), 31.42 (d, *J* = 58.6 Hz).

(1-(((benzyloxy)carbonyl)amino)-3-phenylpropyl)phosphinic acid *rac*-**10**

(1-amino-3-phenyl-propyl)phosphinic acid **5** (20.5 g, 103 mmol) and Na_2_CO_3_ (24.0 g, 226 mmol) were dissolved in water (300 mL) under argon (required around 15 min for complete dissolution). Then, the solution was cooled to 0 °C and a solution of Cbz-OSu (28.2 g, 113 mmol) in THF (300 mL) was dropwise added to the stirred reaction mixture (over 15 min). The resulting reaction mixture became white and cloudy upon stirring. The reaction was allowed to stir at room temperature under argon for 24 h. The reaction mixture was diluted with water (300 mL) and washed with ethyl acetate. The aqueous layer diluted with ice (~ 300 mL) and then ~ 5 M HCl aq. (300 mL) was added to the stirred solution. A white solid precipitated. The resulting mixture was stirred for 15 min and then allowed to stand for 3 hours to allow complete precipitation. The product was isolated by filtration to afford the product as a white solid in quantitative yield and taken directly through to the next step. See data for *R*-**10**.

(*S*)-1-phenylethan-1-aminium (*R*)-(1-(((benzyloxy)carbonyl)amino)-3-phenylpropyl)phosphinate **S1**

(1-(benzyloxycarbonylamino)-3-phenyl-propyl)phosphinic acid **10** (38.0 g, 114 mmol) was suspended in EtOH (250 mL) and the mixture was refluxed until completely dissolved, then (1*S*)-1-phenylethanamine (0.40 mL, 114 mmol) was added dropwise. The resulting clear yellow solution was stirred at reflux for 15 min and then the heating was removed. When the solution had cooled to room temperature it was transferred into a conical flask and placed in the refrigerator (5 °C). After 1 h at 5 °C, white crystals began to form on the bottom of the conical flask. After 24 h, the flask was gently shaken and left to stand at 5 °C for a further 72 h. The white crystals formed were isolated by filtration (32 g). The material was re-dissolved in EtOH (150 ml) and heated until completely dissolved. The resulting orange solution was allowed to cool to room temperature and placed in the refrigerator (5 °C) for four days. The resulting white solid was filtered and washed with cold ethanol to afford a white solid (20.6 g). This material was subjected to a further crystallization cycle. 20.6 g was dissolved in absolute EtOH (70 ml) and the resulting white suspension was heated to reflux until complete dissolution. The clear solution was allowed to slowly cool to room temperature (at first by switching off the heat with stirring for 30 min, then removing from the heating block and allowing the reaction mixture to stand for a further 30 min). One tip of a spatula (~ 2 mg) of batch 1 crystals were added to this solution as seeds. The flask was transferred to the refrigerator (5 °C) and allowed to stand for 18 h. The precipitate was collected by filtration to afford thin white crystals (10.0 g, 19%). **[[α]^22^_D_ = -22.161 (T=22.4 ºC; c=1.00 EtOH, l=0.5 dm), lit. [α]^20^_D_ = -22.6]**^[[3]](#endnote-3)^

(1-(((benzyloxy)carbonyl)amino)-3-phenylpropyl)phosphinic acid **10**

(*S*)-1-phenylethan-1-aminium (*R*)-(1-(((benzyloxy)carbonyl)amino)-3-phenylpropyl)phosphinate **S1** (10.0 g, 22.0 mmol) was dissolved in an aqueous solution of 4M HCl aq. (110 mL, 440 mmol) and the clear solution allowed to stir at room temperature for 3 h. Upon stirring, the solution started to become cloudy and a fine white precipitate began to form. The precipitate was isolated by filtration to afford a white solid (9.5 g). However, this material still contained traces of amine salt (as determined by ^1^H NMR). Therefore, the solid was re-dissolved in 50 mL of 4M HCl aq. and allowed to stir for a further 3 h. The precipitate was filtered and washed with distilled water (4 x 50 mL) to afford a white solid (6.88 g, 94%) [α]^20^_D_ = -36.35 [average of two measurements, (T=20 ºC; c=1.00 EtOH, l=0.05 dm) c.f. literature data: [α]_D_ = -35.7 (T=20 ºC; c=1.00 EtOH]. See data for *R*-**10**.^iii^

By chiral HPLC (Method A) this material was determined to be a 5.8:1 mixture of enantiomers.

This material was used as the precursor for synthesis of compounds **3**, **14**, **17**, **18**, **19**.

Scheme 2: Route B, Preparation of **10**

f) CuSO_4_, DCM, 20 ^o^C, 87%; g) Rb_2_CO_3_, DCM, 20 ^o^C, 56%; h) 4M HCl aq., reflux, 93%; i) Cbz-OSu, Na_2_CO_3_, THF/H_2_O, 94%; j) (*S*)-(-)-α-methylbenzylamine, EtOH, multiple recrystallizations; k) 4M HCl aq., 20 ^o^C, 33% over two steps.

(*S*)-2-Methyl-*N*-(3-phenylpropylidene)propane-2-sulfinamide **8**

(*S*)-2-Methyl-*N*-(3-phenylpropylidene)propane-2-sulfinamide **8** was prepared based on the method by Yao et al.^[[4]](#endnote-4)^ 2-Methylpropane-2-sulfinamide **7** (3.04 g, 25.1 mmol) was dissolved in CH_2_Cl_2_ (50 mL) and CuSO_4_ (6.40 g, 40.1 mmol) was added. 3-Phenylpropanal (8.1 mL, 50.2 mmol) was added dropwise to the stirred solution. The reaction mixture was stirred at room temperature under argon for four hours. The reaction mixture was then filtered through a short pad of silica gel, eluting with ethyl acetate. The filtrate was concentrated and purified by column chromatography (Biotage, SNAP KP-Sil 100g, 5-40% ethyl acetate in cyclohexane). The product was isolated as clear, colorless oil (5.19 g, 87%). HRMS (ESI^+^): calcd for C_13_H_20_NOS (M + H)^+^ 238.1260, found 238.1263. ^1^H NMR (500 MHz, CDCl_3_) δ 8.16 (t, *J* = 4.3 Hz, 1H), 7.38 – 7.29 (m, 3H), 7.25 – 7.21 (m, 2H), 3.02 (dd, *J* = 7.8, 6.0 Hz, 2H), 2.94 – 2.88 (m, 2H), 1.17 (s, 9H). ^13^C NMR (126 MHz, CDCl_3_) δ 168.72, 140.45, 128.68, 128.46, 126.40, 56.74, 37.60, 31.51, 22.39.

Ethyl (1*R*)-1-((tert-butylsulfinyl)amino)-3-phenylpropyl (diethoxymethyl)phosphinate **9**

Ethyl (1*R*)-1-((*tert*-butylsulfinyl)amino)-3-phenylpropyl (diethoxymethyl)phosphinate **9** was prepared based on the method reported by Yao et al.^iv^ Rb_2_CO_3_ (1.51 g, 6.53 mmol) was suspended in CH_2_Cl_2_ (10 mL) and ethyl 1,1-(diethoxymethyl)-*H*-phosphinate (Ciba-Geigy reagent, 992 mg, 5.06 mmol) was added. The resulting reaction mixture was stirred at room temperature under argon for 30 minutes. Then (*S*)-2-Methyl-*N*-(3-phenylpropylidene)propane-2-sulfinamide **8** was added. The resulting reaction mixture was stirred at room temperature under argon overnight. After which time, LC-MS analysis indicated full conversion. On multi-gram scale the reaction was stirred for up to four days until full conversion was achieved. The reaction mixture was then quenched with water. The layers were separated and the aqueous layer was extracted with ethyl acetate (x 2). The combined organic layers were washed with brine, dried over sodium sulfate and concentrated under reduced pressure. The crude product was purified by column chromatography (Biotage, SNAP KP-Sil, 10 g, 0-80% ethyl acetate in cyclohexane). The product was isolated as clear colorless oil (306 mg, 56%). HRMS (ESI^+^): calcd for C_20_H_37_NO_5_PS (M + H)^+^ 434.2125, found 434.2126. ^1^H NMR (500 MHz, CDCl_3_) Major diastereoisomer: δ 7.31 – 7.27 (m, 2H), 7.24 – 7.16 (m, 3H), 5.09 (dd, *J* = 18.9, 10.2 Hz, 1H), 4.32 – 4.22 (m, 2H), 3.96 – 3.76 (m, 4H), 3.73 – 3.69 (m, 2H), 2.91 (ddd, *J* = 14.5, 10.3, 5.0 Hz, 1H), 2.74 – 2.66 (m, 1H), 2.36 – 2.15 (m, 1H), 2.00 – 1.87 (m, 1H), 1.34 (t, *J* = 7.1 Hz, 3H), 1.28 (s, 9H), 1.26 – 1.23 (m, 6H).

((*R*)-1-amino-3-phenylpropyl)phosphinic acid *R*-**5**

Ethyl (1*R*)-1-((tert-butylsulfinyl)amino)-3-phenylpropyl (diethoxymethyl)phosphinate **9** (5.06 g, 11.7 mmol) was dissolved in 4 M HCl (60 mL, 240 mmol) and the resulting solution was refluxed for 2 h 40 min. The reaction mixture was then cooled to room temperature and washed with CH_2_Cl_2_ (x 3). The aqueous layer was concentrated. The remaining residue was re-suspended in ethanol (15 mL) and propylene oxide (350 mL) was added. Upon addition of propylene oxide, the solids initially completely dissolved before the formation of a white precipitate. The mixture was cooled to -20ºC for three hours to allow complete precipitation. The product was isolated by filtration and washed with diethyl ether (white solid, 2.16 g, 93%). HRMS and NMR data matched the data obtained for (*RS*)-**5**.

1-((((benzyloxy)carbonyl)amino)-3-phenylpropyl)phosphinic acid **10**

(1-Amino-3-phenylpropyl)phosphinic acid *R*-**5** (2.05 g, 10.3 mmol), sodium carbonate (2.42 g, 22.8 mmol) and water (10 mL) were added to a round bottom flask under argon. The resulting mixture was stirred at room temperature under argon for 30 minutes until all solids were finely suspended and the reaction mixture was a white cloudy suspension. The reaction mixture was then cooled to 0 ºC and a solution of *N*-(benzyloxycarbonyloxy)succinimide (2.89 g, 11.6 mmol) in THF (10 mL) was added portion-wise to the reaction mixture over 5 minutes. The resulting reaction mixture was stirred at 0 ºC for 25 minutes and then at room temperature for 69 hours. The reaction mixture was diluted with water and washed with ethyl acetate. The aqueous layer was diluted with ice and acidified by addition of 1 M aq. HCl. The product precipitated from the solution and was isolated by filtration and washed with diethyl ether (white solid, 3.21 g, 94%). HRMS (ESI+): calcd for C_17_H_21_NO_4_P (M + H)^+^ 334.1203, found 334.1203. NMR data matched *R*-**10**. Analysis by chiral chromatography (Method A) showed this material was an 8.4:1 mixture of enantiomers.

Whilst this reaction usually worked cleanly, on a larger scale a side product was sometimes observed resulting from rearrangement of the CBz group onto phosphorus. The reasons for formation of the side product are unclear. In this case the side product could be removed by recrystallization as described below.

((1*R*)-1-(benzyloxycarbonylamino)-3-phenyl-propyl)phosphinic acid ***R*-10**

1-(((Benzyloxy)carbonyl)amino)-3-phenylpropyl)phosphinic acid (e.r. 8.4:1, 3.84 g, 11.5 mmol) was suspended in EtOH (100 mL) and the mixture was refluxed until completely dissolved. Then, (1*S*)-1-phenylethanamine (1.49 mL, 11.5 mmol) was added as a solution in EtOH (2 mL, then 0.5 mL to rinse flask) dropwise and the resulting clear solution was allowed to stir at reflux for 15 min. The reaction mixture was allowed to cool to room temperature and the solution concentrated in vacuo. The resulting residue was taken up in EtOH (50 ml) and heated to reflux. EtOH was then added portion-wise until all the solids had dissolved (375 ml). The reaction mixture was allowed to cool slowly to room temperature overnight and then cooled to 5 °C in a refrigerator for a further 4 hours. A fluffy white solid was collected by filtration (1.84 g) which corresponded to a regioisomeric side product of the required material (see previous step) and was discarded. The filtrate was collected and concentrated in vacuo to afford the required product as an off-white solid (3.19 g). This solid was dissolved in refluxing EtOH (30 mL) to afford a pale yellow solution which was allowed to cool slowly to room temperature and then cooled to 5 °C in a refrigerator overnight. The resulting solid was collected by filtration, washed with a small portion of Et_2_O and dried in vacuo to afford the required product as a white solid (1.87 g). This material was taken directly through to the next step to free the salt.

(*S*)-1-phenylethan-1-aminium (*R*)-(1-(((benzyloxy)carbonyl)amino)-3-phenylpropyl)phosphinate **S1** (1.85 g, 4.07 mmol) was dissolved in an aqueous solution of 4M HCl aq. (20.7 mL, 82.8 mmol). Initially a gummy precipitate was observed, but over time a fine white precipitate began to form. The suspension was periodically sonicated to facilitate formation of the suspension. The reaction was allowed to stir for 4 h. The precipitate was isolated by filtration, then washed with water (x 3), followed by a small portion of Et_2_O and dried under vacuum to afford a white solid (1.25 g, 33% over two steps). HRMS (ESI+): calcd for C_17_H_20_NNaO_4_P (M + Na)^+^ 356.1022, found 356.1021. ^1^H NMR (500 MHz, DMSO-*d*_6_) δ 7.71 (d, *J* = 8.8 Hz, 1H), 7.38 (d, *J* = 4.4 Hz, 4H), 7.35 – 7.30 (m, 1H, 0.5H, PH), 7.27 (t, *J* = 7.4 Hz, 2H), 7.21 – 7.14 (m, 3H), 6.24 (s, 0.5H, PH), 5.14 – 5.01 (m, 2H), 3.55 (ddd, *J* = 13.0, 9.8, 5.5 Hz, 1H), 2.72 (ddd, *J* = 13.8, 9.1, 5.0 Hz, 1H), 2.59 – 2.51 (m, 1H), 1.96 – 1.87 (m, 1H), 1.85 – 1.75 (m, 1H). ^13^C NMR (126 MHz, DMSO-*d*_6_) δ 156.27, 141.10, 137.02, 128.43, 128.39, 128.35, 127.88, 127.74, 125.93, 65.66, 49.89 (d, *J* = 105.3 Hz), 31.36 (d, *J* = 12.4 Hz), 28.34 (d, *J* = 3.7 Hz). ^31^P NMR (202 MHz, DMSO-d6) δ 27.77. Analysis by chiral chromatography (Method A) RT = 5.26 min, >95%, er >99:1.

This material was used as the precursor for synthesis of compounds **1** (DG013A) and **2** (DG013B).

Figure 1. Comparison of enantiopurities of **10** obtained using different preparation methods.


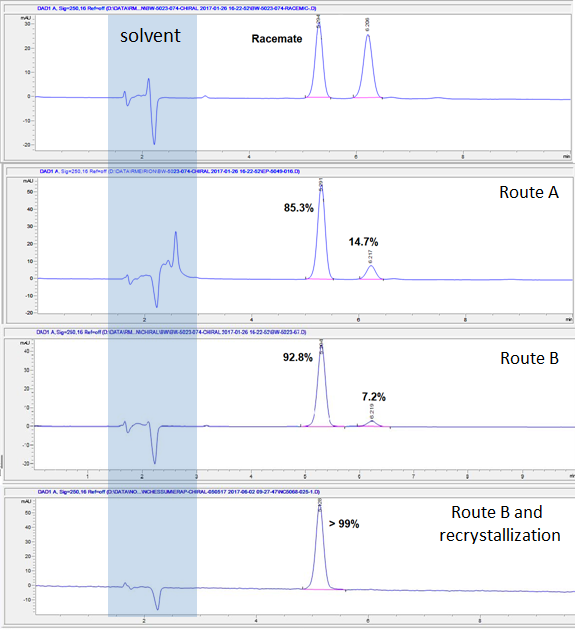


Conditions: Column: Chiralpak QD-AX, 150 x 4.6 mm, solvents, MeOH/AcOH/NH_4_OAc = 98/2/0.5 (v/v/w), isocratic, flow rate 1 mL/min, sample concentration: 1 mg/mL in methanol.

Scheme 3: Final steps to prepare DG013A **1** and DG013B **2.**

l) BSA, 20 ^o^C, 16 h; m) NaOH, EtOH, H_2_O, 20 ^o^C, 24 h, 80% (over 2 steps); n) 33% HBr in AcOH, 88%; o) Boc_2_O, Et_3_N, DMF, 78%; p) EDC.HCl, HOBt, DIPEA, DCM, 20 ^o^C, 80%; q) TFA/DCM/TIS/H_2_O, quant.; r) Separation by HPLC, DG013A **1** 31%, DG013B **2** 24%

Table 1; Optimization of the reaction conditions for the Phospha-Michael addition

| Entry | Conditions | Product*  (%) | Reference |
| --- | --- | --- | --- |
| 1 | **10** (1.0 eq.), DIPEA (4.5 eq.), -78 °C, TMSCl (4.5 eq.), RT, 2 h; then -78 °C, **11** (1.1 eq.), then warm to RT. | 20 | *J. Organomet. Chem.* **2002**, *646*, 212-222. |
| 2 | **10** (1.0 eq.), DIPEA (7.0 eq.), **11** (1.1 eq.), -78 °C, TMSCl (7.0 eq.), then warm to RT. | 11 | *Org. Lett.* **2009**, *11* (20), 4696-4699. |
| 3 | **10** (1.0 eq.), *N*,*O*-bis(trimethylsilyl)acetamide (7.0 eq.), **11** (1.3 eq.), RT. | 100 | *J. Med. Chem.* **2000**, *43* (7), 1398-1408. |
| 4 | **10** (1.0 eq.), 1,1,1,3,3,3-Hexamethyldisilazane (13.0 eq.), 110 °C; then 90 °C, **11** (1.3 eq.). | 60 | *J. Med. Chem.* **2016**, *59* (19), 9107-9123.  Angew. Chem., Int. Ed. **2007**, *46*, 3275-3277. |

* As determined by LCMS analysis

2-((((*R*)-1-(((benzyloxy)carbonyl)amino)-3-phenylpropyl)(hydroxy)phosphoryl)methyl)-4-methylpentanoic acid **15**

The procedure for the Phospha-Michael addition was adapted from the method reported by Chen et al.^[[5]](#endnote-5)^ ((1*R*)-1-(benzyloxycarbonylamino)-3-phenyl-propyl)phosphinic acid **10** (500 mg, 1.50 mmol) was dissolved in *N,O*-bis(trimethylsilyl)acetamide (BSA, 1.32 mL, 5.38 mmol) and ethyl 4-methyl-2-methylene-pentanoate in cyclohexane **11** (325 mg, 2.08 mmol) was added. The resulting reaction mixture was heated to 35ºC and was stirred under argon for three hours and then at room temperature for 65 hours. After which time, LC-MS analysis indicated full conversion. The reaction was quenched by addition of water and was extracted with ethyl acetate (x 4). The combined organic layers were washed with brine and dried over sodium sulfate and concentrated (colorless oil, solidifies upon standing). The crude product (**13**) was used in the next synthetic step without further purification.

((1*R*)-1-(benzyloxycarbonylamino)-3-phenyl-propyl)-(2-ethoxycarbonyl-4-methyl-pentyl)phosphinic acid **13** (734 mg, 1.50 mmol) was dissolved in 10 mL ethanol. Then, 2 M NaOH (4.00 mL, 8.00 mmol) was added and the resulting reaction mixture was heated to 35 ºC and was stirred under argon for 48 hours. After which time, LC-MS analysis indicated full conversion. The reaction mixture was concentrated in vacuo and the resulting residue diluted with water and acidified by addition of 2 M HCl. The aqueous layer was then extracted with ethyl acetate (x 4). The combined organic layers were washed with brine, dried over sodium sulfate and concentrated in vacuo to afford the product **S6** (white solid, 555 mg, 80%) as 1:1 mixture of diastereomers (as indicated by ^1^H NMR). HRMS (ESI+): calcd for C_24_H_33_NO_6_P (M + H)^+^ 462.2040, found 462.2040.  ^1^H NMR (500 MHz, CD_3_OD-*d*_4_) δ 7.45 – 7.28 (m, 5H), 7.27 – 7.11 (m, 5H), 5.21 – 5.09 (m, 2H), 3.85 (dddd, *J* = 18.2, 11.7, 8.7, 2.8 Hz, 1H), 2.80 (dtt, *J* = 13.9, 9.0, 4.1 Hz, 2H), 2.59 (dtd, *J* = 13.5, 8.5, 4.4 Hz, 1H), 2.11 (ddd, *J* = 15.3, 12.4, 8.4 Hz, 2H), 1.93 – 1.82 (m, 1H), 1.75 (dddd, *J* = 21.1, 15.7, 12.0, 5.0 Hz, 1H), 1.56 (ddt, *J* = 17.3, 8.5, 5.1 Hz, 2H), 1.43 – 1.27 (m, 1H), 0.92 (d, *J* = 6.4 Hz, 3H), 0.88 (dd, *J* = 6.4, 2.4 Hz, 3H).

(2*R*)-2-((((*R*)-1-amino-3-phenylpropyl)phosphoryl)methyl)-4-methylpentanoic acid **S2**

2-((((*R*)-1-(((benzyloxy)carbonyl)amino)-3-phenylpropyl)(hydroxy)phosphoryl)methyl)-4-methylpentanoic acid **15** (153 mg, 0.332 mmol) was added to a round bottom flask under argon and cooled to 0 ºC. Then, HBr in acetic acid (2.0 mL, 35 mmol) was added. The resulting reaction mixture was stirred at 0 ºC under argon for 10 minutes and then at room temperature for two hours. The reaction mixture was then diluted with diethyl ether and concentrated. The crude product was recrystallized from ethyl acetate/cyclohexane. The product was isolated by filtration as the hydrobromide salt (white solid, 120 mg, 88%). HRMS (ESI+): calcd for C_16_H_27_NO_4_P (M + H)^+^ 328.1672, found 328.1671. ^1^H NMR (500 MHz, CD_3_OD) δ 7.35 – 7.24 (m, 4H), 7.24 – 7.16 (m, 1H), 3.41 (td, *J* = 8.2, 5.4 Hz, 1H), 2.95 – 2.84 (m, 2H), 2.79 (ddd, *J* = 13.7, 10.5, 5.9 Hz, 1H), 2.42 – 2.19 (m, 2H), 2.10 – 1.99 (m, 1H), 1.91 (td, *J* = 15.6, 4.0 Hz, 1H), 1.66 (ddd, *J* = 14.9, 12.9, 6.5 Hz, 2H), 1.52 – 1.38 (m, 1H), 0.95 (dd, *J* = 17.3, 6.2 Hz, 6H).

(*2R*)-2-(((((*R*)-1-(tert-butoxycarbonyl)amino)-3-phenylpropyl)(hydroxyl)phosphoryl)methyl)-4-methylpentanoic acid **S3**

(*2R*)-2-((((*R*)-1-amino-3-phenylpropyl)phosphoryl)methyl)4-methylpentanoic acid hydrobromide salt **S2** (624 mg, 1.53 mmol) and triethylamine (1.07 mL, 7.65 mmol) were dissolved in anhydrous DMF (18 mL) and di tert-butyl dicarbonate (500 mg, 2.29 mmol) was added. The resulting reaction mixture was stirred at room temperature under argon for 3 days. The reaction mixture was diluted with ethyl acetate and 1M HCl aq. The layers were separated and the organic layer was washed with 1M HCl aq. (x 2), water and brine. The organic layer was dried over sodium sulfate and concentrated to afford the crude product as a pale yellow oil. Purification by reverse phase Biotage chromatography (30-100% MeOH in H_2_O + 0.1% formic acid) afforded the product as a pale yellow solid (512 mg, 78%). HRMS (ESI^+^): calcd for C_17_H_27_NO_6_P (M + H, -^t^Bu)^+^ 372.1571 found 372.1572. ^1^H NMR (500 MHz, CD_3_OD) δ 7.26 (t, *J* = 7.4 Hz, 2H), 7.20 (d, *J* = 8.0 Hz, 2H), 7.16 (t, *J* = 7.2 Hz, 1H), 3.80 (dddd, *J* = 20.5, 11.6, 8.6, 2.9 Hz, 1H), 2.82 (dtt, *J* = 18.2, 9.4, 4.8 Hz, 2H), 2.60 (qd, *J* = 10.2, 8.6, 3.3 Hz, 1H), 2.13 (ddd, *J* = 19.9, 10.0, 6.6 Hz, 2H), 1.81 (dddd, *J* = 23.0, 19.8, 11.4, 5.6 Hz, 2H), 1.60 (tq, *J* = 14.5, 7.5, 7.1 Hz, 2H), 1.488 (s, 9H^A^), 1.485 (s, 9H^B^), 1.45 – 1.43 (m, 1H), 0.94 (app dd, *J* = 6.3, 4.4 Hz, 3H^A^+3H^B^), 0.91 (d, *J* = 6.3 Hz, 3H^A+B^). ^13^C NMR (126 MHz, CD_3_OD) δ 178.79, 157.99, 142.45, 142.39, 129.62, 129.45, 127.07, 80.76, 80.73, 50.96 (d, *J* = 77.3 Hz), 50.11 (d, *J* = 77.3 Hz), 44.85 (d, *J* = 10.7 Hz), 44.27 (d, *J* = 8.7 Hz), 38.52 (dd, *J* = 7.1, 3.5 Hz), 33.24 (t, *J* = 12.6 Hz), 30.50 (t, *J* = 20.9 Hz), 29.69 (d, *J* = 19.6 Hz), 28.77, 28.55, 27.18, 27.15, 23.49, 23.45, 22.31. (*N*-*Boc rotamers + diastereomers visible in NMR spectra*).

((*R*)-2-(((*S*)-1-amino-3-(*1H*-indol-3-yl)-1-oxopropan-2-yl)carbamoyl)-4-methylpentyl)(((*R*)-1-(tert-butoxycarbonyl)amino)-3-phenylpropyl)phosphinic acid **S4**

2-amino-2-(*1H*-indol-3-yl)acetamide hydrochloride (344 mg, 1.52 mmol), HOBt (174 mg, 1.28 mmol) , and EDC. HCl (1.12 g, 6.15 mmol) were dissolved in DCM (9.0 mL) and DIPEA (0.75 mL, 4.28 mmol) .Then, (2*S*)-2-((((1*R*)-1-(*tert*-butoxycarbonylamino)-3-phenyl-propyl)-hydroxy-phosphoryl)methyl)-4-methyl-pentanoic acid **S3** (501 mg, 1.17 mmol) was added and the resulting reaction mixture was stirred at room temperature under argon in a sealed flask. The reaction was stirred at room temperature for 2 days. The reaction mixture was concentrated and the resulting residue was partitioned between ethyl acetate and 1M HCl. The layers were separated and the organic layer was washed with 1M HCl (x 3), water and brine. The organic layer was dried over sodium sulfate and concentrated in vacuo. The crude product was purified by reversed-phase Biotage chromatography (30-100% MeOH/H_2_O + 0.1% formic acid) to afford the product as an off-white solid (574 mg, 80%). HRMS (ESI+): calcd for C_32_H_46_N_4_O_6_P (M + H)^+^ 613.3149, found 613.3142. ^1^H NMR (500 MHz, CD_3_OD) δ 7.62 (t, *J* = 7.7 Hz, 1H^A+B^), 7.31 (dd, *J* = 8.1, 4.0 Hz, 1H^A+B^), 7.26 (t, *J* = 7.4 Hz, 2H^A+B^), 7.18 (dd, *J* = 20.1, 7.2 Hz, 3H^A+B^), 7.10 (br s, 1H^A+B^), 7.09 – 7.04 (m, 1H^A+B^), 7.02 – 6.97 (m, 1H^A+B^), 4.78 (dd, *J* = 11.5, 3.8 Hz, 1H^B^), 4.64 (t, *J* = 7.4 Hz, 1H^A^), 3.91 – 3.79 (m, 1H^B^), 3.75 (ddd, *J* = 11.7, 8.8, 2.9 Hz, 1H^A^), 3.54 (dd, *J* = 14.5, 3.6 Hz, 1H^B^), 3.35 – 3.28 (m, 1H^A+B^), 3.16 (dd, *J* = 14.6, 8.0 Hz, 1H^A+B^), 2.99 (td, *J* = 15.2, 11.4 Hz, 1H^B^), 2.81 (ddd, *J* = 13.4, 8.8, 4.4 Hz, 1H^A+B^), 2.76 – 2.68 (m, 1H^A+B^), 2.60 (dt, *J* = 15.8, 8.0 Hz, 1H^A+B^), 2.17 – 2.05 (m, 1H^A+B^), 2.05 – 1.90 (m, 1H^A+B^), 1.89 – 1.73 (m, 1H^A+B^), 1.67 – 1.53 (m, 1H^A+B^), 1.49 (s, 9H^B^), 1.48 (s, 9H^A^), 1.46 (br s, 2H^B^), 1.35 – 1.21 (m, 2H^A^), 0.97 – 0.86 (m, 1H^A+B^), 0.77 (dd, *J* = 35.9, 6.2 Hz, 6H^A^), 0.54 – 0.44 (m, 6H^B^).

((*R*)-2-(((*S*)-1-amino-3-(1*H*-indol-3-yl)-1-oxopropan-2-yl)carbamoyl)-4-methylpentyl)((*R*)-1-amino-3-phenylpropyl)phosphinic acid **1** and ((*S*)-2-(((S)-1-amino-3-(*1H*-indol-3-yl)-1-oxopropan-2-yl)carbamoyl)-4-methylpentyl)((*R*)-1-amino-3-phenylpropyl)phosphinic acid **2**

The solvent mixture was prepared according to Kokkala^[[6]](#endnote-6)^ using TFA 9.6 ml: DCM 9.8 ml: 0.4 ml TIS: 0.2 ml H_2_O. ((2*S*)-2-(((1*S*)-2-amino-1-(*1H*-indol-3-ylmethyl)-2-oxo-ethyl)carbamoyl)-4-methyl-pentyl)-((1*R*)-1-(tert-butoxycarbonylamino)-3-phenyl-propyl)phosphinic acid **S4** (563 mg, 0.918 mmol) was dissolved in the solvent mix (12 mL) and the reaction mixture left to stir at room temperature for 2 h. After which time, the reaction was complete as monitored by LCMS. The reaction mixture was concentrated in vacuo, the resulting residue was re-dissolved in EtOAc/cyclohexane, concentrated again and dried under high vacuum to afford the crude product as a pale brown foam (659 mg). A portion of the crude product (180 mg) was purified by preparative HPLC (Method **B**) using an isocratic gradient 20% solvent A in solvent B (solvent A = 10% MeCN in 90% H_2_O + 0.1% formic acid, solvent B = 10% H_2_O in 90% MeCN + 0.1% formic acid). To afford good separation it was necessary to dissolve the crude product in 75:25 solvent A:B for injection onto the column and to load the compound batch-wise (5 mg aliquots). This yielded DG013A (**1**) as a pale yellow solid (40.6 mg, 8.5% yield, 31% if all crude had been used) and DG013B (**2**) as an off-white solid (31.1 mg, 6.6%, 24 % if all crude had been used). However, the sample of DG013B was only 92% pure as determined by chiral HPLC (Method A) therefore this material was re-purified under the same column conditions to afford a pure sample as an off-white solid (9.2 mg, 7% if all crude had been used).

((*R*)-2-(((*S*)-1-amino-3-(1*H*-indol-3-yl)-1-oxopropan-2-yl)carbamoyl)-4-methylpentyl)((*R*)-1-amino-3-phenylpropyl)phosphinic acid DG013A **1**

HRMS (ESI^+^): calcd for C_27_H_38_N_4_O_4_P (M+H^+^) 513.2625, found 513.2609. ^1^H NMR (500 MHz, CD_3_OD ) δ 7.62 (d, *J* = 7.8 Hz, 1H), 7.30 (d, *J* = 8.0 Hz, 1H), 7.29 – 7.24 (m, 2H), 7.22 (d, *J* = 7.0 Hz, 2H), 7.17 (t, *J* = 7.1 Hz, 1H), 7.13 (s, 1H), 7.07 (t, *J* = 7.4 Hz, 1H), 6.99 (t, *J* = 7.4 Hz, 1H), 4.63 – 4.57 (m, 1H), 3.41 – 3.34 (m, 1H), 3.20 (dd, *J* = 14.5, 9.4 Hz, 1H), 2.97 – 2.88 (m, 1H), 2.83 (td, *J* = 13.4, 12.0, 4.3 Hz, 1H), 2.25 – 2.12 (m, 1H), 1.99 – 1.82 (m, 2H), 1.60 (t, *J* = 14.7 Hz, 1H), 1.41 – 1.32 (m, 1H), 1.28 – 1.12 (m, 2H), 0.77 (br s, 3H), 0.69 (br s, 3H)^. 13^C NMR (126 MHz, CD_3_OD ) δ 178.47, 176.94, 142.06, 138.09, 129.61, 129.44, 128.71, 127.32, 124.59, 122.38, 119.77, 119.39, 112.31, 111.40, 55.57, 51.65 (d, *J* = 88.0 Hz), 44.98 (d, *J* = 10.8 Hz), 40.74, 33.56 (d, *J* = 6.4 Hz), 32.71 (d, *J* = 94.8 Hz), 31.45, 28.24, 26.58, 23.20, 22.44.^31^P NMR (202 MHz, CD_3_OD) δ 29.81.

((*S*)-2-(((*S*)-1-amino-3-(1*H*-indol-3-yl)-1-oxopropan-2-yl)carbamoyl)-4-methylpentyl)((*R*)-1-amino-3-phenylpropyl)phosphinic acid DG013B **2**

HRMS (ESI^+^): calcd for C_27_H_38_N_4_O_4_P (M+H^+^) 513.2625, found 513.2609. ^1^H NMR (600 MHz, CD_3_OD ) δ 8.50 (br s, 1H, formate), 7.62 (d, *J* = 7.9 Hz, 1H), 7.32 (d, *J* = 8.1 Hz, 1H), 7.29 – 7.24 (m, 2H), 7.24 (d, *J* = 7.0 Hz, 2H), 7.17 (t, *J* = 7.1 Hz, 1H), 7.12 (s, 1H), 7.08 (t, *J* = 7.2 Hz, 1H), 6.99 (t, *J* = 7.4 Hz, 1H), 4.76 (dd, *J* = 11.3, 3.7 Hz, 1H), 3.53 (dd, *J* = 14.8, 3.5 Hz, 1H), 3.01 (dd, *J* = 14.7, 11.5 Hz, 1H), 2.99 – 2.93 (m, 1H), 2.82 (td, *J* = 12.7, 12.0, 5.0 Hz, 1H), 2.74 – 2.62 (m, 2H), 2.24 – 2.16 (m, 1H), 1.98 – 1.81 (m, 2H), 1.45 (t, *J* = 15.0 Hz, 1H), 1.26 (ddd, *J* = 13.9, 11.2, 6.1 Hz, 1H), 0.90 (ddd, *J* = 13.5, 9.5, 5.1 Hz, 1H), 0.64 (tt, *J* = 12.7, 6.4 Hz, 1H), 0.55 – 0.49 (m, 6H). ^13^C NMR (151 MHz, CD_3_OD ) δ 178.67, 177.74, 142.18, 138.33, 129.57, 129.43, 128.47, 127.26, 124.58, 122.39, 119.76, 119.39, 112.27, 111.42, 55.21, 45.74 (d, *J* = 13.9 Hz), 39.61 (d, *J* = 2.6 Hz), 33.69 (d, *J* = 8.1 Hz), 33.13, 31.67, 28.60, 26.19, 23.54, 22.01. ^31^P NMR (202 MHz, CD_3_OD) δ 30.17.

Figure 2. Showing chiral purity of DG013A **1** and DG013B **2** as determined by chiral HPLC (Method A)


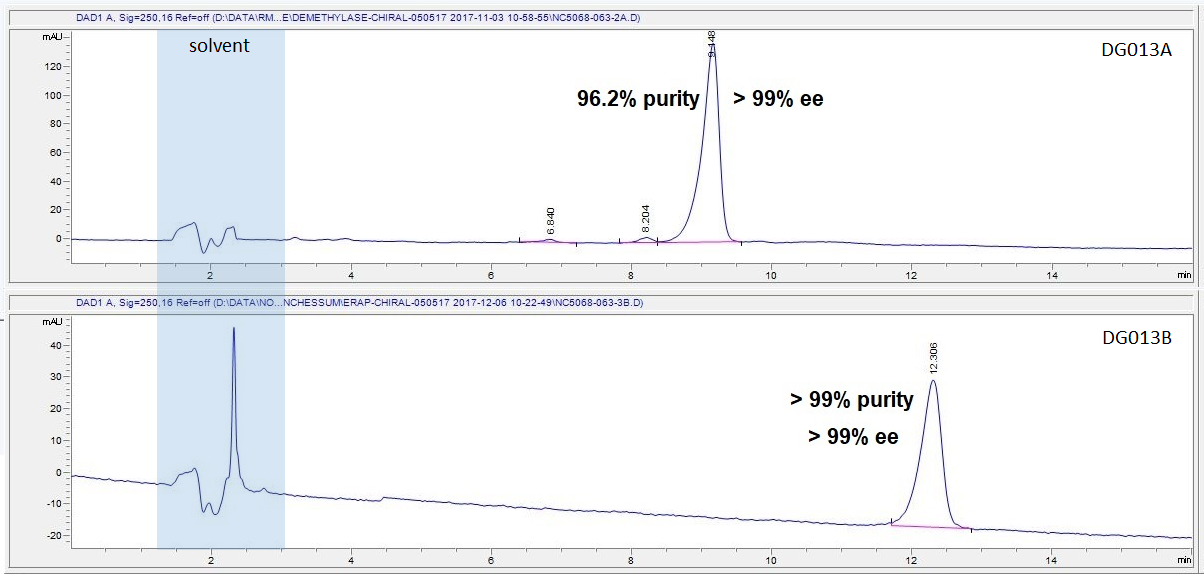


Conditions: Column: Column: Chiralpak QD-AX, 150 x 4.6 mm, solvents, MeOH/AcOH/NH_4_OAc = 98/2/0.5 (v/v/w), isocratic, flow rate 1 mL/min, sample concentration: 1 mg/mL in methanol. To afford good separation, it was necessary to dissolve the crude product in 75:25 solvent A:B for injection onto the column and to load the compound batch-wise (5 mg aliquots).

Scheme 4: Stereoselective Phospha-Michael addition

s) LiOH aq., EtOH, RT, 72 h; t) i) Pivaloyl chloride, NMM; ii) nBuLi, (*R*)-4-benzyloxazolidinone, 27% over 3 steps; u) DIPEA, TMSCl, 0 ^o^C to RT, 20 h; v) Separation by HPLC **14** 38%; w) H_2_O_2_, LiOH aq., 0 ^o^C, 87%

(*R*)-4-benzyl-3-(4-methyl-2-methylenepentanoyl)oxazolidin-2-one **12**

Ethyl 4-methyl-2-methylene-pentanoate **S7** (8.00 g, 35.3 mmol), 1M LiOH aq. solution (70.6 mL, 70.6 mmol) and EtOH (35mL) were combined and allowed to stir at room temperature for 48 h. The organic solvents were removed under reduced pressure. The aqueous layer was acidified to pH 1-3 by dropwise addition of 3M HCl aq. and then extracted with EtOAc (4 x 100 mL). The combined organic layer was dried (Na_2_SO_4_), filtered and concentrated under reduced pressure. The crude 4-methyl-2-methylenepentanoic acid **S8** was used directly in the next step. ^1^H NMR (500 MHz, Chloroform-*d*) δ 7.73 (br s, 1H), 6.28 (d, *J* = 1.6 Hz, 1H), 5.59 (s, 1H), 2.17 (dd, *J* = 7.1, 1.0 Hz, 2H), 1.82 (dp, *J* = 13.5, 6.8 Hz, 1H), 0.89 (d, *J* = 6.7 Hz, 6H).

(4*R*)-4-benzyloxazolidin-2-one (5.00 g, 28.2 mmol) was dissolved in anhydrous THF (50 ml) and the resulting clear solution was cooled to -78 °C. Then, n-BuLi (1.6 M in hexanes, 18.8 mL, 28.2 mmol) was added dropwise and the resulting yellow/orange suspension was allowed to stir for 1 h at -78 °C under argon. 4-Methyl-2-methylenepentanoic acid **S8** (4.34 g, 33.8 mmol) was dissolved in anhydrous THF in a separate flask and the solution was cooled to -78 °C. Then, *N*-methylmorpholine (3.72 mL, 33.8 mmol) was added dropwise, followed by the dropwise addition of trimethylacetyl chloride (4.17 mL, 33.8 mmol). The reaction mixture was allowed to stir for 1 h at -78 °C under argon. After 1 h, the two mixtures were combined. The acryloyl mixed anhydride was transferred into the flask containing the oxazolidinone via a 20 mL syringe with a wide bore oven-dried needle. The resulting mixture was allowed to stir for 0.5 h at -78 °C, then the cooling bath was removed, the reaction was allowed to warm to room temperature and left to stir for a further 1 h. The reaction mixture was diluted with DCM (100 mL) and quenched with sat. NH_4_Cl aq. (100 mL). The aqueous layer was extracted with DCM (3 x 100 mL) and the combined organic layers were washed with brine (100 mL), dried (Na_2_SO_4_), filtered and concentrated under reduced pressure to afford the crude product as a thick pale yellow oil ( ~6 g). The crude product was purified by two rounds of Biotage chromatography (0-15% EtOAc/cyclohexane) to afford the product as a white solid (2.15 g, 26.5%). HRMS (ESI^+^): calcd for C_17_H_22_NO_3_ (M+H^+^) 288.1594, found: 288.1594. ^1^H NMR (500 MHz, CDCl_3_) δ 7.34 (ddd, *J* = 7.5, 6.3, 1.3 Hz, 2H), 7.30 – 7.26 (m, 1H), 7.24 – 7.20 (m, 2H), 5.47 (s, 1H), 5.44 – 5.43 (m, 1H), 4.71 (dddd, *J* = 9.5, 8.0, 4.5, 3.5 Hz, 1H), 4.28 – 4.22 (m, 1H), 4.17 (dd, *J* = 9.0, 4.5 Hz, 1H), 3.38 (dd, *J* = 13.4, 3.4 Hz, 1H), 2.80 (dd, *J* = 13.4, 9.5 Hz, 1H), 2.37 (ddd, *J* = 14.3, 6.7, 0.9 Hz, 1H), 2.28 – 2.20 (m, 1H), 1.83 – 1.69 (m, 1H), 0.96 (dd, *J* = 6.6, 4.2 Hz, 6H). ^13^C NMR (126 MHz, CDCl_3_) δ 171.17, 152.96, 143.16, 135.27, 129.56, 129.12, 127.55, 121.44, 66.54, 55.55, 42.75, 37.88, 27.60, 22.63, 22.34

((*S*)-2-((*R*)-4-benzyl-2-oxooxazolidine-3-carbonyl)-3-methylbutyl)((*R*)-1-(((benzyloxy)carbonyl)amino)-3-phenylpropyl)phosphinic acid (major product, **14**) and ((*R*)-2-((*R*)-4-benzyl-2-oxooxazolidine-3-carbonyl)-3-methylbutyl)((*R*)-1-(((benzyloxy)carbonyl)amino)-3-phenylpropyl)phosphinic acid (minor product, **S11**).

Based on the procedure described by Ebetino et al.^[[7]](#endnote-7)^ Phosphinic acid **10*** (1.38 g, 4.14 mmol) was suspended in anhydrous DCM (30mL), the reaction mixture was cooled to 0 °C and DIPEA (3.17 mL, 18.2 mmol) was added dropwise (upon addition of the base the mixture immediately became a pale yellow clear solution), followed by the dropwise addition of chlorotrimethylsilane (15.8 mL, 124 mmol). The reaction mixture was allowed to stir at room temperature for 3 h under inert atmosphere. Then, oxazolidinone **S7** (1.43 g, 4.97 mmol) was added in one portion and the reaction mixture was allowed to stir at room temperature over which time the reaction mixture became a pale yellow solution. After 48 h, the reaction mixture was cooled to -10 °C and 40 mL of ethanol was added and the resulting mixture was allowed to stir for 30 min. The reaction mixture was concentrated under reduced pressure to afford the crude product as a thick orange oil. The crude material was purified by preparative HPLC (Method C) to afford the required product **14** as a white glassy solid (977 mg, 38%) and the epimeric minor product **S11**, also as a glassy white solid (334 mg, 13%).

((*S*)-2-((*R*)-4-benzyl-2-oxooxazolidine-3-carbonyl)-3-methylbutyl)((*R*)-1-(((benzyloxy)carbonyl)amino)-3-phenylpropyl)phosphinic acid (major product, **14**).

HRMS (ESI^+^): calcd for C_34_H_42_N_2_O_7_P (M+H^+^) 621.2724, found: 621.2722. 1H NMR (500 MHz, CD_3_OD ) δ 7.45 – 7.12 (m, 15H), 5.21 – 5.09 (m, 2H), 4.71 (tt, *J* = 8.3, 3.2 Hz, 1H), 4.33 – 4.18 (m, 3H), 3.84 (t, *J* = 9.7 Hz, 1H), 3.12 (dd, *J* = 13.4, 3.4 Hz, 1H), 2.90 (dd, *J* = 13.5, 8.5 Hz, 1H), 2.79 (ddd, *J* = 13.7, 8.9, 4.8 Hz, 1H), 2.60 (dt, *J* = 13.6, 8.3 Hz, 1H), 2.26 (dt, *J* = 15.4, 10.7 Hz, 1H), 2.12 (d, *J* = 10.7 Hz, 1H), 1.93 – 1.84 (m, 2H), 1.61 (q, *J* = 6.9 Hz, 2H), 1.39 – 1.32 (m, 1H), 0.94 (dd, *J* = 21.9, 5.9 Hz, 6H).

((*R*)-2-((*R*)-4-benzyl-2-oxooxazolidine-3-carbonyl)-3-methylbutyl)((*R*)-1-((((benzyloxy)carbonyl)amino)-3-phenylpropyl)phosphinic acid (minor product, **S11**).

^1^H NMR (500 MHz, CD_3_OD ) δ 7.45 – 7.15 (m, 15H), 5.24 – 5.09 (m, 2H), 4.71 – 4.62 (m, 1H), 4.38 (s, 1H), 4.29 – 4.20 (m, 1H), 4.17 (dd, *J* = 8.9, 2.4 Hz, 1H), 4.03 (ddd, *J* = 11.6, 8.4, 3.1 Hz, 1H), 3.38 – 3.36 (m, 1H), 2.86 – 2.72 (m, 2H), 2.69 – 2.56 (m, 1H), 2.31 (td, *J* = 14.4, 10.7 Hz, 1H), 2.17 (ddt, *J* = 15.9, 12.8, 6.4 Hz, 1H), 1.95 – 1.81 (m, 2H), 1.57 (td, *J* = 6.5, 3.3 Hz, 2H), 1.42 – 1.25 (m, 1H), 0.94 – 0.88 (m, 6H).

* To conduct these studies, the phosphinic acid precursor used was obtained via Route A. Therefore, this starting material was a 5.8:1 mixture of enantiomers at the chiral center adjacent to phosphorus. Whilst it was possible to separate the diastereomers formed from the Phospha-Michael addition by HPLC, it was impossible to separate all four diastereomers, i.e. the 5.8:1 mix at the first chiral center was carried through.

(2*R*)-2-((((*R*)-1-amino-3-phenylpropyl)(hydroxy)phosphoryl)methyl)-4-methylpentanoic acid **S12**

Based on the procedure described by Evans et al;^[[8]](#endnote-8)^ oxazolidinone **14** (977mg, 1.57 mmol) was dissolved in THF (24 mL) and the resulting solution was stirred under argon at 0 °C. Then hydrogen peroxide (2.14 mL, 6.30 mmol) was added followed by an aqueous solution of LiOH (75.4mg, 3.15 mmol, in 8 mL of distilled water) and the reaction mixture was stirred at 0 °C for 1 h. The reaction was quenched by addition of 5 mL of sat. Na_2_SO_3_ aq. and 5 mL of sat. NaHCO_3_ aq. The mixture was partially concentrated under reduced pressure. 10 mL of distilled water were added. The aqueous layer was extracted with DCM (3 x 5 mL), then the aqueous layer was acidified to pH 1 by addition of 3M HCl aq. and extracted with EtOAc (4 x 5 mL). The combined organic layers were dried over Na_2_SO_4_, filtered and concentrated under reduced pressure to afford the crude product as a white solid. The crude material was subjected to trituration with 100 mL of ether, using sonication to ensure the solid was properly suspended, then was allowed to stand for 1 h; after which, the solid was isolated by filtration and dried under vacuum (632 mg, 87%).* HRMS (ESI^+^) calcd for C_24_H_33_NO_6_P (M+H^+^) 462.2045, found: 462.2040. ^1^H NMR (500 MHz, Methanol-*d*_4_) δ 7.44 – 7.29 (m, 5H), 7.29 – 7.10 (m, 5H), 5.15 (d, *J* = 1.2 Hz, 2H), 3.84 (ddd, *J* = 11.8, 8.9, 3.0 Hz, 1H), 2.81 (ddt, *J* = 13.8, 8.9, 5.1 Hz, 2H), 2.60 (dt, *J* = 13.6, 8.2 Hz, 1H), 2.18 – 2.07 (m, 2H), 1.94 – 1.85 (m, 1H), 1.84 – 1.71 (m, 1H), 1.58 (tdd, *J* = 17.5, 8.1, 5.7 Hz, 2H), 1.45 – 1.35 (m, 1H), 0.91 (dd, *J* = 16.7, 6.4 Hz, 6H). ^13^C NMR (126 MHz, MeOD) δ 178.79, 158.65, 142.26, 138.31, 129.62, 129.51, 129.44, 129.08, 128.93, 127.07, 68.32, 51.57 (d, *J* = 105.6 Hz), 44.34 (d, *J* = 11.1 Hz), 38.52 (d, *J* = 5.6 Hz), 33.14 (d, *J* = 11.1 Hz), 30.65, 30.22 (d, *J* = 88.7 Hz), 27.14, 23.36, 22.32. ^31^P NMR (202 MHz, MeOD) δ 48.14. * As highlighted in the previous step, this material was an inseparable mixture (5:8:1 in favor of the required product) of enantiomers at the first chiral center adjacent to phosphorus.

Synthesis of (2*S*)-2-((((1*S*)-2-amino-1-(1*H*-indol-3-ylmethyl)-2-oxo-ethyl)carbamoyl)-4-methyl-pentyl)-((1*R*)-1-(benzyloxycarbonylamino)-3-phenyl-propyl)phosphinic acid **3**

2-((((*R*)-1-(((benzyloxy)carbonyl)amino)-3-phenylpropyl)(hydroxy)phosphoryl)methyl)-4-methylpentanoic acid **S12** (73.0 mg, 0.158 mmol), HOBt (28.0 mg, 0.207 mmol), and *N*-(3-dimethylaminopropyl)-*N’*-ethylcarbodiimide hydrochloride (EDC, 164 mg, 0.856 mmol) were dissolved in dichloromethane (0.40 mL) and *N,N*-diisopropylethylamine (0.10 mL, 0.59 mmol). Then, 2-amino-2-(1*H-*indol-3-yl)acetamide hydrochloride (46.4 mg, 0.206 mmol) was added and the resulting reaction mixture was stirred at room temperature under argon overnight. After this time, the reaction mixture was concentrated and the resulting residue was re-dissolved in ethyl acetate and washed sequentially with ~1M aq. HCl (x 2), water (x 1) and brine. The organic layer was dried over sodium sulfate and concentrated. The crude product was purified by reversed-phase Biotage chromatography (Biotage SNAP Ultra C19. Solvent A = 0.1% formic acid in methanol, Solvent B = 0.1% formic acid in water. Gradient of 30%-100% solvent A in solvent B). The product was isolated as white solid (57.9 mg, 57%). The product was further purified by semi-preparative HPLC (Gradient III). The product was isolated as white solid (11.2 mg, 11%). HRMS (ESI^+^): calcd for C_35_H_44_N_4_O_6_P (M + H)^+^ 647.2993, found 647.2978. ^1^H NMR (500 MHz, CD_3_OD) δ 7.63 (dt, *J* = 7.8, 1.0 Hz, 1H), 7.42 – 7.37 (m, 2H), 7.35 (t, *J* = 7.4 Hz, 2H), 7.33 – 7.29 (m, 2H), 7.27 – 7.21 (m, 2H), 7.16 (d, *J* = 7.9 Hz, 3H), 7.11 – 7.05 (m, 2H), 7.03 – 6.97 (m, 1H), 5.20 – 5.07 (m, 2H), 4.66 (dd, *J* = 8.1, 6.6 Hz, 1H), 3.82 (ddd, *J* = 11.6, 8.3, 2.9 Hz, 1H), 3.32 – 3.26 (m, 1H), 3.16 (dd, *J* = 14.6, 8.1 Hz, 1H), 2.80 (td, *J* = 8.6, 4.2 Hz, 1H), 2.72 (s, 1H), 2.64 – 2.50 (m, 1H), 2.13 (td, *J* = 8.4, 5.4 Hz, 1H), 1.95 (ddd, *J* = 15.5, 11.3, 7.8 Hz, 1H), 1.89 (dd, *J* = 12.1, 6.2 Hz, 1H), 1.74 (ddd, *J* = 15.4, 12.6, 6.0 Hz, 1H), 1.49 – 1.34 (m, 1H), 1.26 (ddt, *J* = 15.8, 8.5, 5.1 Hz, 2H), 0.77 (dd, *J* = 31.2, 6.1 Hz, 6H). ^13^C NMR (126 MHz, CD_3_OD) δ 177.03 (d, *J* = 7.7 Hz), 176.65, 158.67 (d, *J* = 4.1 Hz), 142.15, 138.30, 138.04, 129.66, 129.51, 129.46, 129.08, 128.92, 128.78, 127.10, 124.45, 122.37, 119.77, 119.45, 112.25, 111.28, 67.93, 55.19, 51.20 (d, *J* = 106.1 Hz), 44.15 (d, *J* = 8.4 Hz), 39.62 (d, *J* = 3.9 Hz), 33.08 (d, *J* = 11.8 Hz), 30.51, 28.65, 26.78, 23.46, 22.25. ^31^P NMR (202 MHz, CD_3_OD) δ 48.58.

((*R*)-2-(((S)-3-(*1H*-indol-3-yl)-1-methoxy-1-oxopropan-2-yl)carbamoyl)-4-methylpentyl)((*R*)-1-amino-3-phenylpropyl)phosphinic acid **17**

Carboxylic acid **S13*** (50.0mg, 0.117 mmol), HOBt (17.32 mg, 0.128 mmol) and EDC. HCl (117mg, 0.614 mmol) were dissolved in DCM (0.30 mL) and DIPEA (0.07 mL, 0.427 mmol). Then, L-tryptophan methyl ester hydrochloride (38.7 mg, 0.152 mmol) was added and the resulting reaction mixture was stirred at room temperature under argon for 20 h. The reaction mixture was concentrated and the resulting residue was re-dissolved in ethyl acetate (1 mL) and 1M HCl aq. (1 mL). The layers were separated and the organic layer was washed with 1M HCl aq. (3 x 1 mL), water (1 x 1 mL) and brine (1 x 1 mL). The organic layer was dried (Na_2_SO_4_) and concentrated under reduced pressure to afford the crude product as a white solid. This crude material **S14** (46.6 mg) was used directly in the next step.

***S13** was made from **S12** via the same CBz deprotection and *N*-Boc re-protection conditions as used to make **15**.

The solvent mixture was prepared according to Kokkala^iii^ using TFA 9.6 ml: DCM 9.8 ml: 0.4 ml TIS: 0.2 ml H_2_O. Ester **S14** (46.6 mg, 0.074 mmol) was dissolved in 1 mL of the solvent mixture and stirred at room temperature under argon for 2 h. Then, the reaction mixture was concentrated in vacuo. The resulting residue was re-dissolved in ethyl acetate (1 mL) and cyclohexane (1 mL) was added, the resulting mixture was concentrated again to afford the crude product as a colorless thick oil (46.6 mg). The crude product was purified by reversed-phase Biotage chromatography (30-100% MeOH in H_2_O + 0.1% formic acid) to afford the product as a glassy white solid (35.5 mg, 57% over two steps). HRMS (ESI^+^): calcd for C_28_H_39_N_3_O_5_P (M + H)^+^ 528.2622, found 528.2613.^1^H NMR (500 MHz, CD_3_OD ) δ 8.08 (br s, 0.39H, formic acid), 7.54 (dt, *J* = 7.9, 1.1 Hz, 1H), 7.33 – 7.14 (m, 7H), 7.07 (ddd, *J* = 8.2, 7.0, 1.2 Hz, 1H), 7.01 (ddd, *J* = 8.0, 7.0, 1.1 Hz, 1H), 4.66 (dd, *J* = 7.6, 6.4 Hz, 1H), 3.62 (s, 3H), 3.32 – 3.18 (m, 2H), 2.84 – 2.74 (m, 3H), 2.67 – 2.57 (m, 1H), 2.23 – 2.16 (m, 1H), 1.95 – 1.85 (m, 2H), 1.65 – 1.50 (m, 3H), 1.43 – 1.34 (m, 1H), 0.89 (dd, *J* = 23.0, 6.3 Hz, 6H). ^13^C NMR (126 MHz, CD_3_OD) δ 178.73, 173.92, 142.08, 137.96, 129.57, 129.42, 128.64, 127.28, 124.70, 122.42, 119.82, 119.11, 112.37, 110.64, 55.12, 52.56, 52.14 (d, J = 91.3 Hz), 45.70 (d, J = 13.7 Hz), 40.09, 33.61 (d, J = 9.1 Hz), 33.54 (d, J = 95.9 Hz), 32.79, 32.04, 30.99, 28.27, 26.72, 23.27, 22.60. ^31^P NMR (202 MHz, CD_3_OD) δ 30.67.

((2*R*)-2-((((*R*)-1-amino-3-phenylpropyl)(hydroxy)phosphoryl)methyl)-4-methylpentanoyl)-L-tryptophan **18**

((1*R*)-1-amino-3-phenyl-propyl)-((2*S*)-2-(((1*S*)-1-(*1H*-indol-3-ylmethyl)-2-methoxy-2-oxo-ethyl)carbamoyl)-4-methyl-pentyl)phosphinic acid **17** (30.0 mg, 0.057 mmol), 1M LiOH aq. (0.11 mL, 0.114 mmol) and EtOH ( 1.00 mL) were combined and allowed to stir at room temperature overnight. The organic solvents were removed under reduced pressure and the aqueous layer was acidified to pH 3-4 by dropwise addition of 3M aq. HCl. The aqueous layer was extracted with EtOAc (3 x 10 mL). The combined organic layer was dried (Na_2_SO_4_), filtered and concentrated under reduced pressure to afford a pale yellow thick oil. The crude product was purified by reversed-phase Biotage chromatography (30-100% MeOH in water + 0.1% formic acid) to afford the product as a glassy white solid (6.1 mg, 21%). HRMS (ESI^+^, 212 nM): calcd for C_27_H_37_N_3_O_5_P (M + H)^+^ 514.2465, found 514.2455.^1^H NMR (500 MHz, CD_3_OD ) δ 8.08 (s, 0.6H, formic acid), 7.58 (d, *J* = 7.8 Hz, 1H), 7.32 – 7.12 (m, 7H), 7.09 – 6.95 (m, 2H), 4.63 (dd, *J* = 8.4, 5.4 Hz, 1H), 3.38 – 3.30 (m, 1H), 3.22 (dd, *J* = 14.7, 8.4 Hz, 1H), 2.76 (qd, *J* = 12.0, 10.1, 4.6 Hz, 3H), 2.68 – 2.53 (m, 2H), 2.22 – 2.10 (m, 1H), 1.94 – 1.79 (m, 2H), 1.63 – 1.47 (m, 3H), 1.35 (dt, *J* = 12.0, 6.1 Hz, 1H), 0.84 (dd, *J* = 20.5, 6.1 Hz, 5H). ^13^C NMR (126 MHz, CD_3_OD ) δ 178.67 (d, *J* = 5.4 Hz), 175.29, 164.50, 142.13, 137.95, 129.54, 129.42, 128.76, 127.24, 124.68, 122.36, 119.79, 119.23, 112.32, 111.08, 54.97, 51.77 (d, *J* = 91.1 Hz), 44.75 (d, *J* = 10.1 Hz), 40.25, 34.16 (d, *J* = 9.5 Hz), 31.50 (d, *J* = 95 Hz), 30.99, 28.23, 26.68, 23.25, 22.61. ^31^P NMR (202 MHz, CD_3_OD) δ 30.03.

((1*R*)-1-(*tert*-butoxycarbonylamino)-3-phenyl-propyl)-((2*S*)-2-(2-(1*H*-indol-3-yl)ethylcarbamoyl)-4-methyl-pentyl)phosphinic acid **S14**

(2*S*)-2-((((1*R*)-1-(*tert*-butoxycarbonylamino)-3-phenyl-propyl)-hydroxy-phosphoryl)methyl)-4-methylpentanoic acid **S13** (57.6 mg, 0.135 mmol), HOBt (25.7 mg, 0.190 mmol), and *N*-(3-dimethylaminopropyl)-*N’*-ethylcarbodiimide hydrochloride (EDC, 146 mg, 0.761 mmol) were dissolved in dichloromethane (0.35 mL) and *N,N*-diisopropylethylamine (0.09 mL, 0.49 mmol). Then, tryptamine (50.8 mg, 0.311 mmol) was added and the resulting reaction mixture was stirred at room temperature under argon for 24 hours. The reaction mixture was concentrated. The resulting residue was re-dissolved in ethyl acetate and washed sequentially with ~1 M aq. HCl (x 1), water (x 2) and brine. The organic layer was dried over sodium sulfate and concentrated. The crude product was purified by reversed-phase Biotage chromatography (Biotage SNAP Ultra C19. Solvent A = 0.1% formic acid in methanol, Solvent B = 0.1% formic acid in water. Gradient of 30%-100% solvent A in solvent B). The product **S15** was isolated as off-white solid (19.8 mg, 26%). HRMS (ESI^+^): calcd for C_31_H_45_N_3_O_5_P (M + H)^+^ 570.3091, found 570.3096.

((1*R*)-1-amino-3-phenyl-propyl)-((2*S*)-2-(2-(1*H*-indol-3-yl)ethylcarbamoyl)-4-methyl-pentyl)phosphinic acid **19**

The *N*-Boc-deprotection was carried out according to the method reported by Kokkala et al.^iii^ The required solvent mixture was prepared in a round bottom flask under argon: Trifluoroacetic acid (4.8 mL) + dichloromethane (4.9 mL) + triisopropyl silane (0.2 mL) + water (0.1 mL). ((1*R*)-1-(*tert*-butoxycarbonylamino)-3-phenyl-propyl)-((2*S*)-2-(2-(1*H*-indol-3-yl)ethylcarbamoyl)-4-methyl-pentyl)phosphinic acid **S15** (19.8 mg, 0.035 mmol) was dissolved in the solvent mixture (0.5 mL) described above. The resulting reaction mixture was stirred at room temperature under argon for two hours. The reaction mixture was concentrated and the remaining residue was re-dissolved in ethyl acetate and again concentrated. The crude product was purified by reversed-phase chromatography (Biotage SNAP Ultra C19, 12g, Solvent A = 0.1% formic acid in methanol, Solvent B = 0.1% formic acid in water. Gradient of 30%-100% solvent A in solvent B over 14 CV, then 100% solvent B for 3 CV). The product was isolated as off-white solid (6 mg, 37%). HRMS (ESI^+^): calcd for C_26_H_37_N_3_O_3_P (M + H)^+^ 470.2567, found 470.2555. ^1^H NMR (500 MHz, CD_3_OD) δ 7.55 (d, *J* = 7.8 Hz, 1H), 7.32 (d, *J* = 8.1 Hz, 1H), 7.27 – 7.19 (m, 4H), 7.13 (dt, *J* = 6.0, 2.7 Hz, 1H), 7.10 – 7.03 (m, 2H), 6.99 (ddd, *J* = 8.0, 7.0, 1.0 Hz, 1H), 3.59 (dt, *J* = 13.2, 7.5 Hz, 1H), 3.26 (dt, *J* = 13.1, 7.2 Hz, 1H), 2.94 (dt, *J* = 12.0, 7.3 Hz, 2H), 2.88 – 2.77 (m, 2H), 2.73 – 2.63 (m, 2H), 2.24 (td, *J* = 8.7, 3.7 Hz, 1H), 2.01 – 1.84 (m, 2H), 1.65 – 1.44 (m, 3H), 1.44 – 1.38 (m, 1H), 0.86 – 0.77 (m, 6H). ^13^C NMR (126 MHz, CD_3_OD) δ 178.56 (d, *J* = 4.9 Hz), 141.97, 138.20, 129.58, 129.45, 128.72, 127.29, 123.50, 122.30, 119.56, 119.31, 113.05, 112.23, 51.59 (d, *J* = 90.3 Hz), 45.25 (d, *J* = 11.8 Hz), 41.26, 40.54, 33.52 (d, *J* = 8.1 Hz), 32.57, 30.93, 26.86, 26.21, 23.38, 22.47. ^31^P NMR (202 MHz, CD_3_OD) δ 29.87.

**NMR Spectra and LCMS Traces of Final Compounds**

NMR spectra DG013A **1**: ^1^H NMR (500 MHz, Methanol-*d*_4_), ^13^C NMR (126 MHz, Methanol-*d*_4_), ^31^P NMR (202 MHz, Methanol-*d*_4_) and LC trace


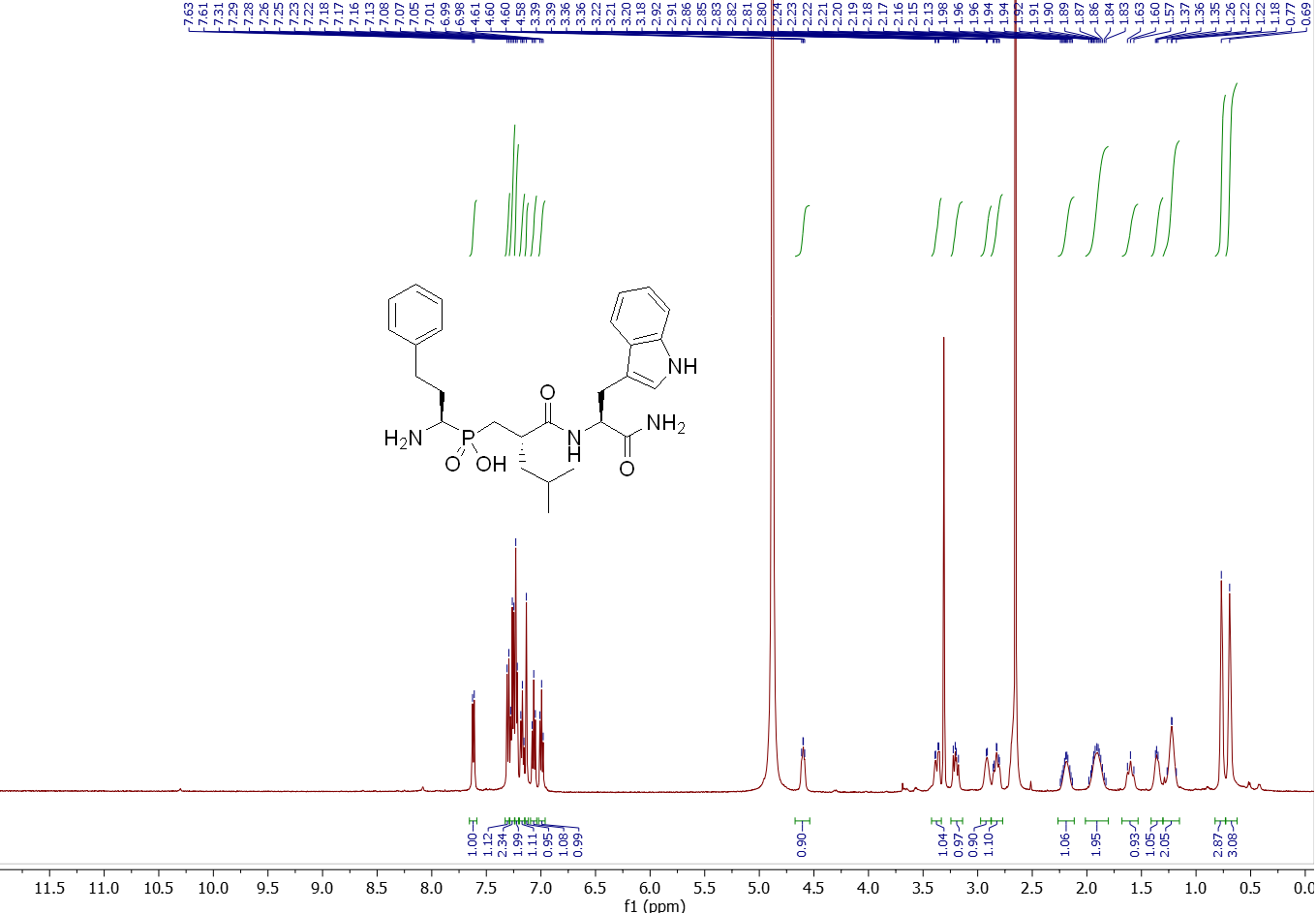


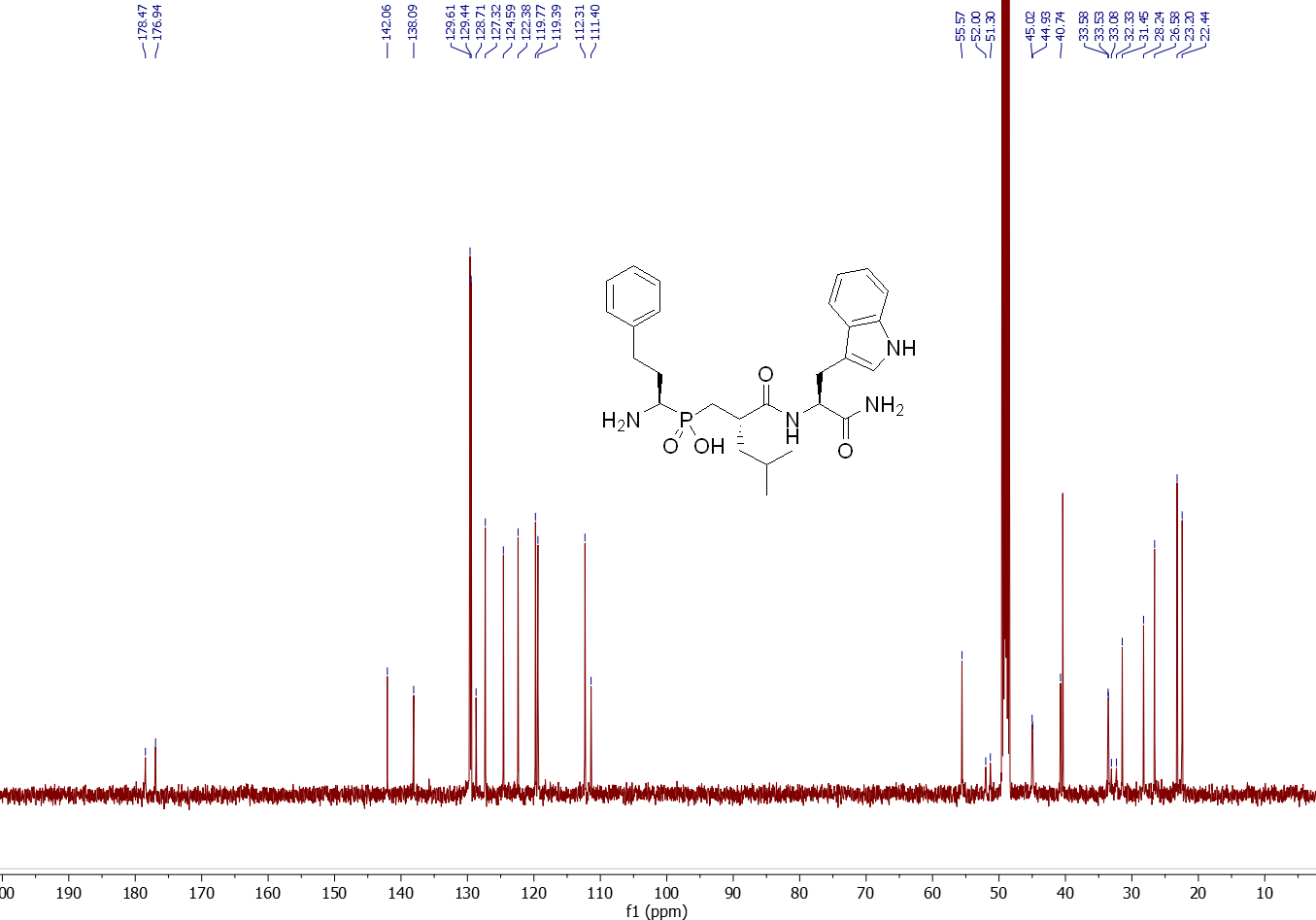


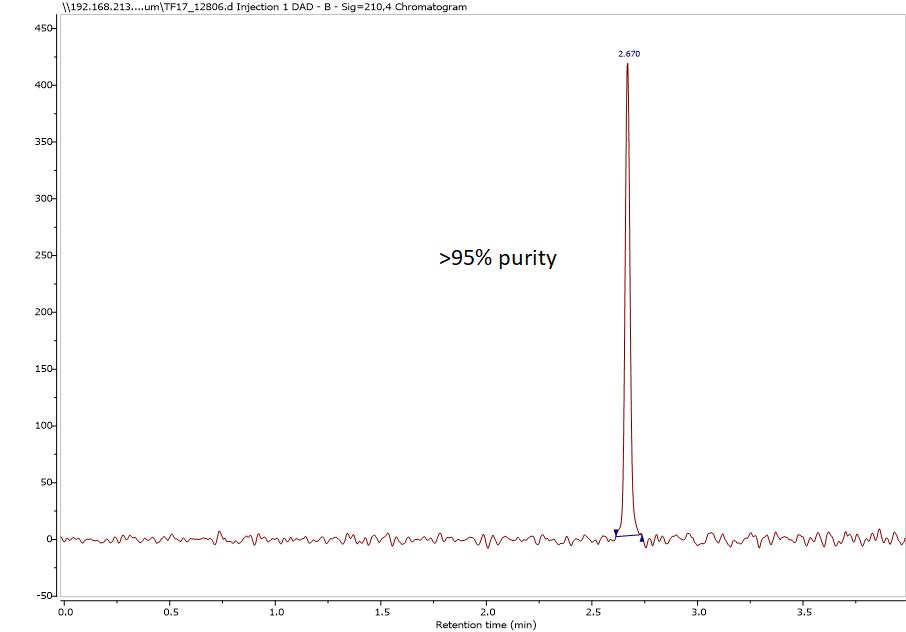

NMR spectra DG013B **2**: ^1^H NMR (500 MHz, Methanol-*d*_4_), ^13^C NMR (126 MHz, Methanol-*d*_4_),^31^P NMR (202 MHz, Methanol-*d*_4_) and LC trace


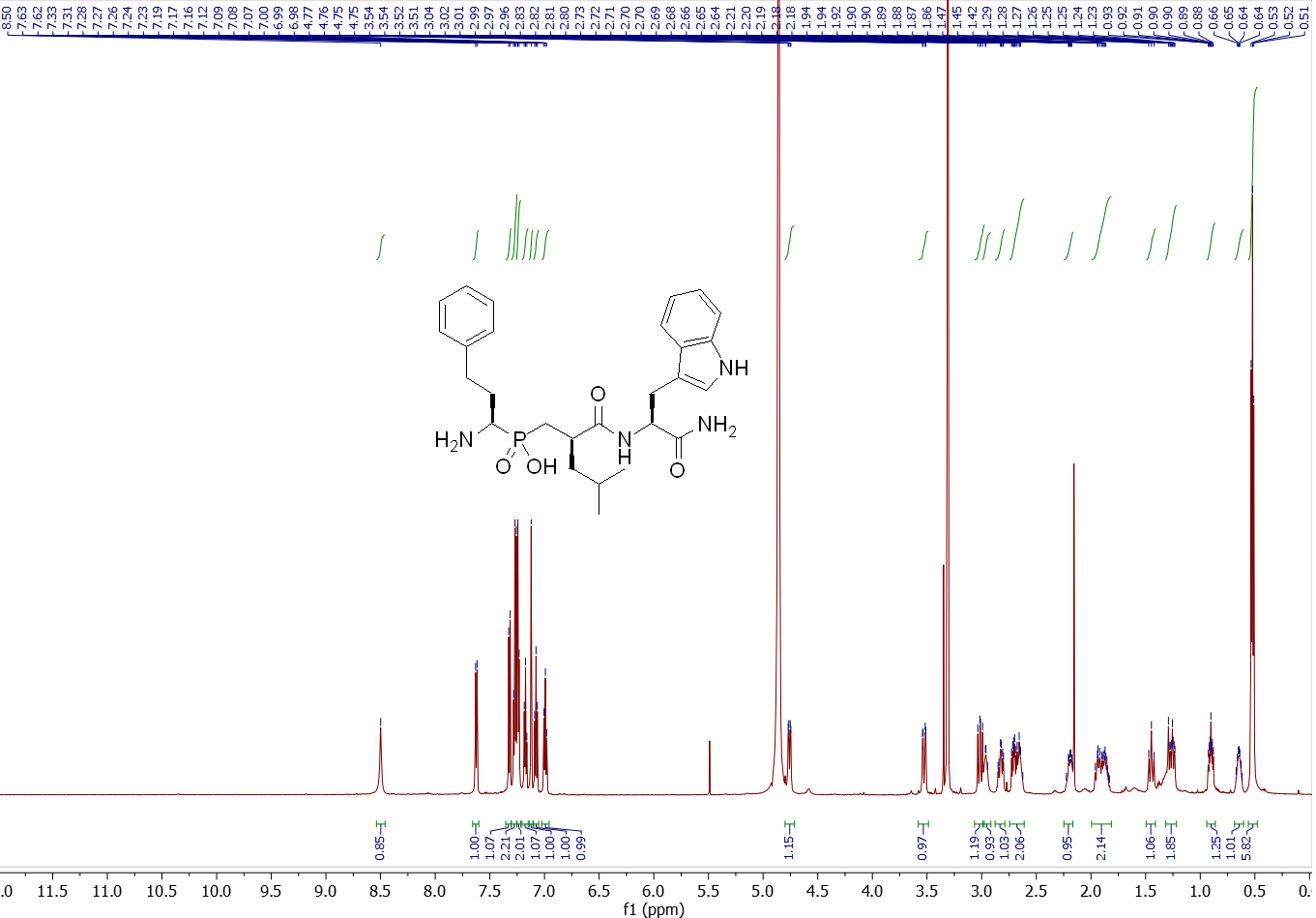


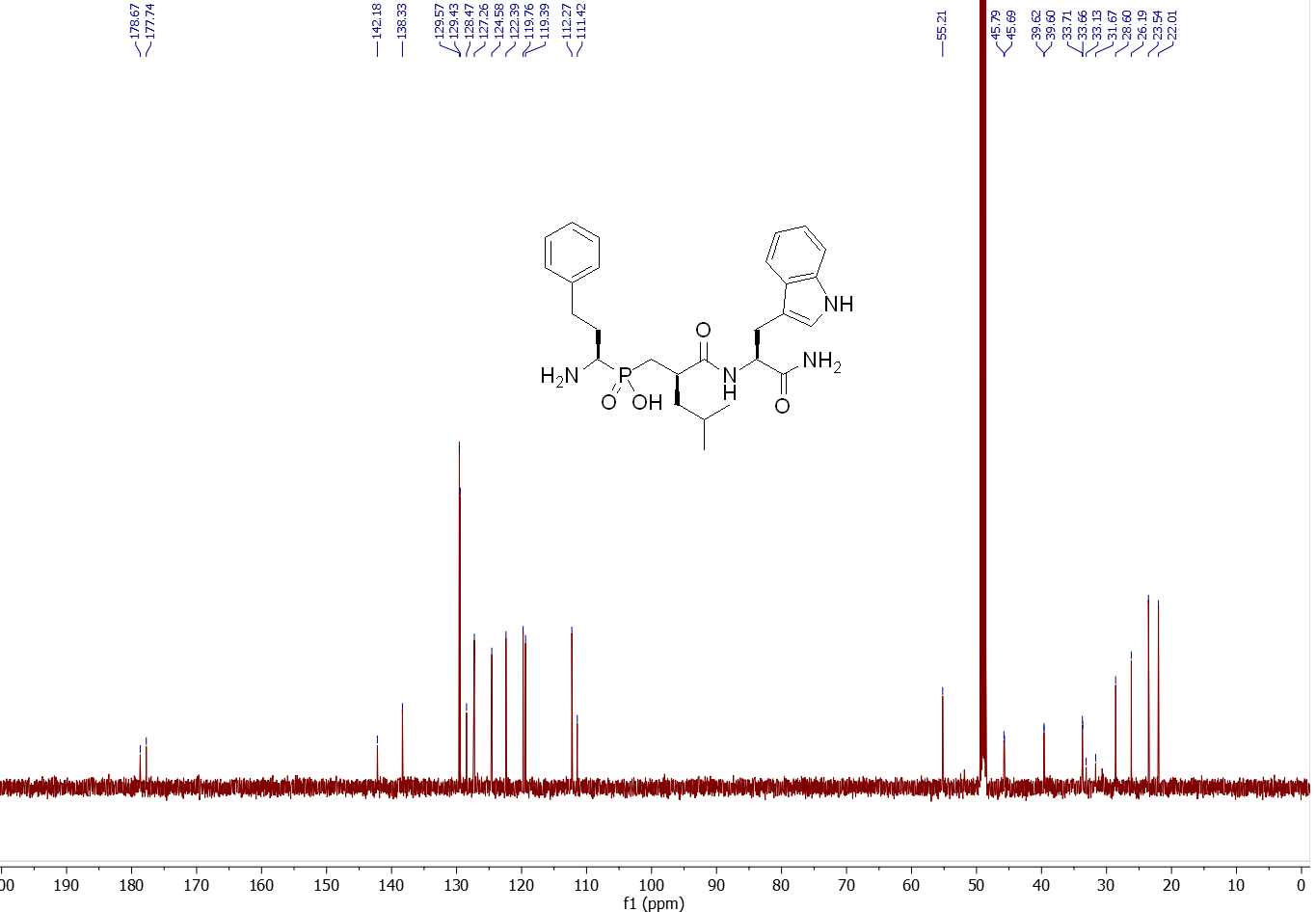


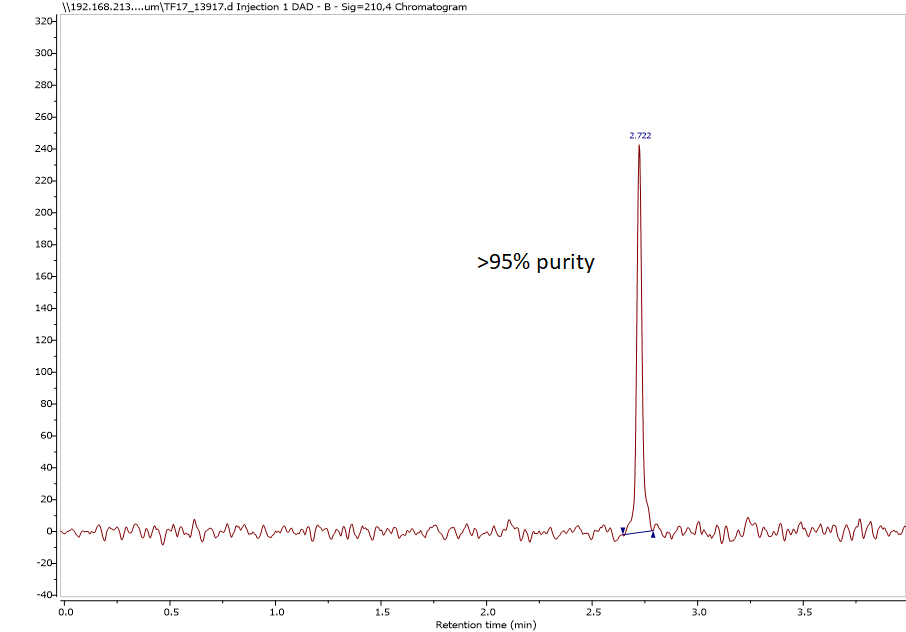

NMR spectra **3**: ^1^H NMR (500 MHz, Methanol-*d*_4_), ^13^C NMR (126 MHz, Methanol-*d*_4_), ^31^P NMR (202 MHz, Methanol-*d*_4_) and mass chromatogram


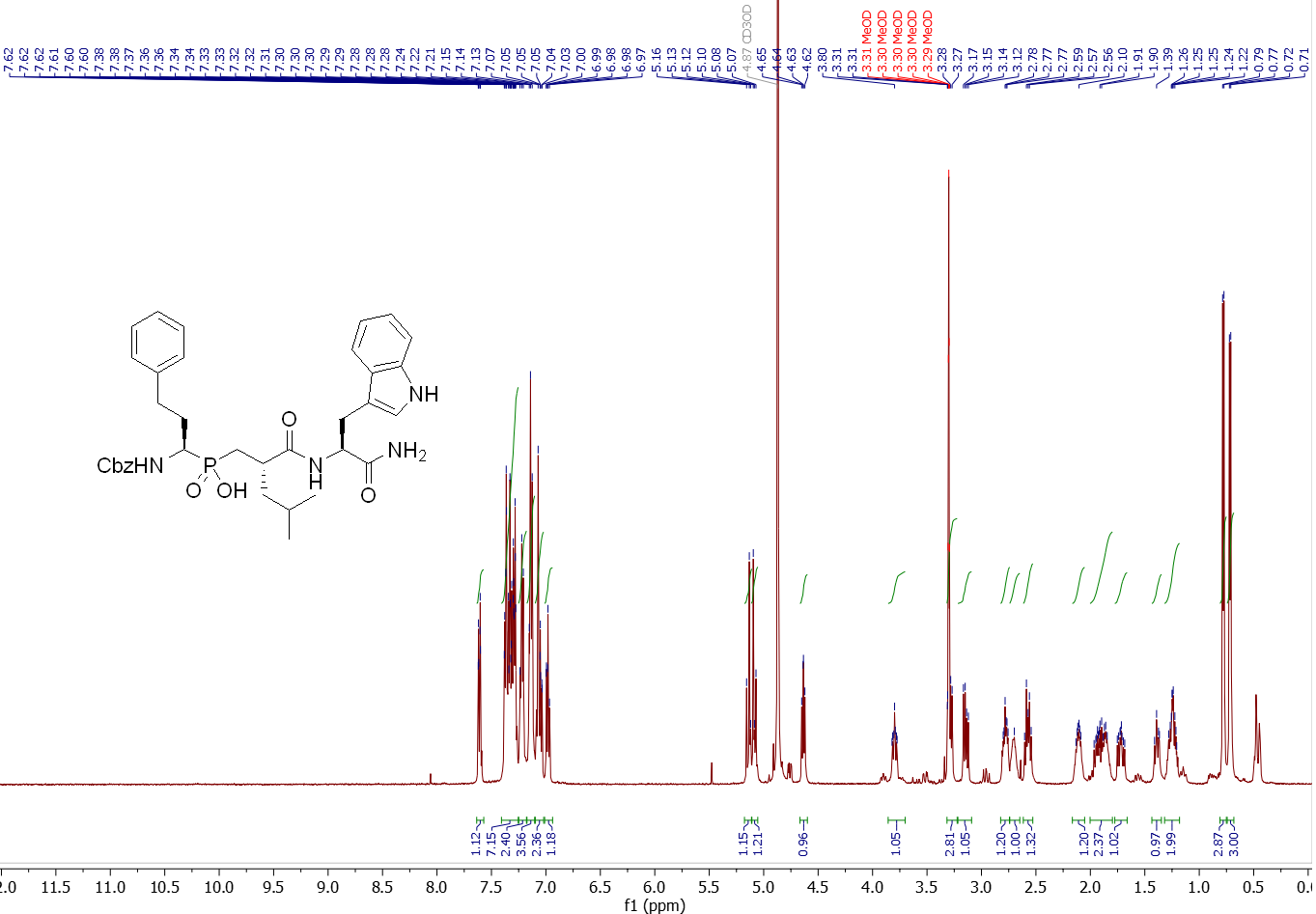


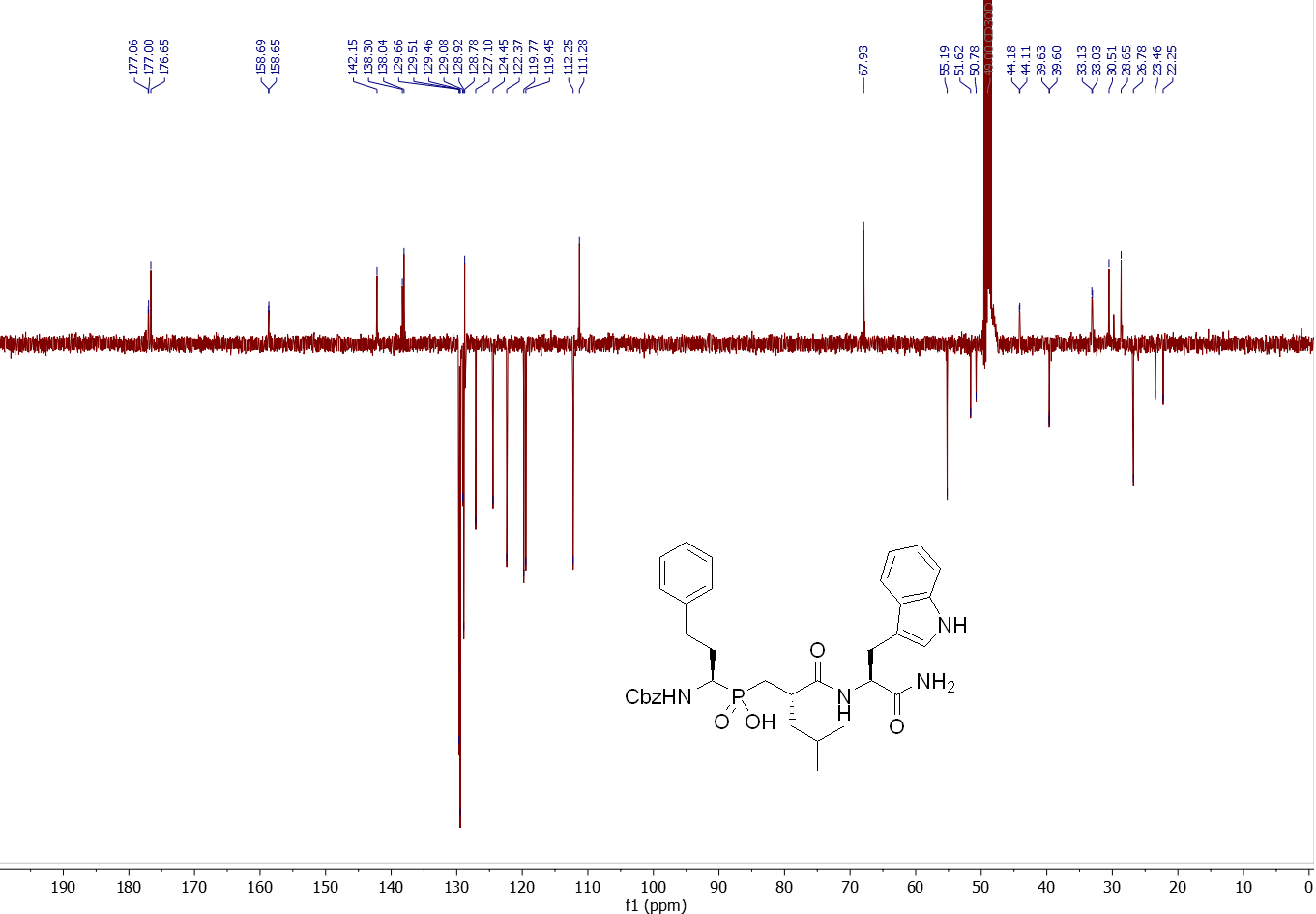


Owing to the poor chromophore of compound 3, it was not possible to obtain a measure of UV purity.

NMR spectra **17**: ^1^H NMR (500 MHz, Methanol-*d*_4_), ^13^C NMR (126 MHz, Methanol-*d*_4_), ^31^P NMR (202 MHz, Methanol-*d*_4_) and LC trace


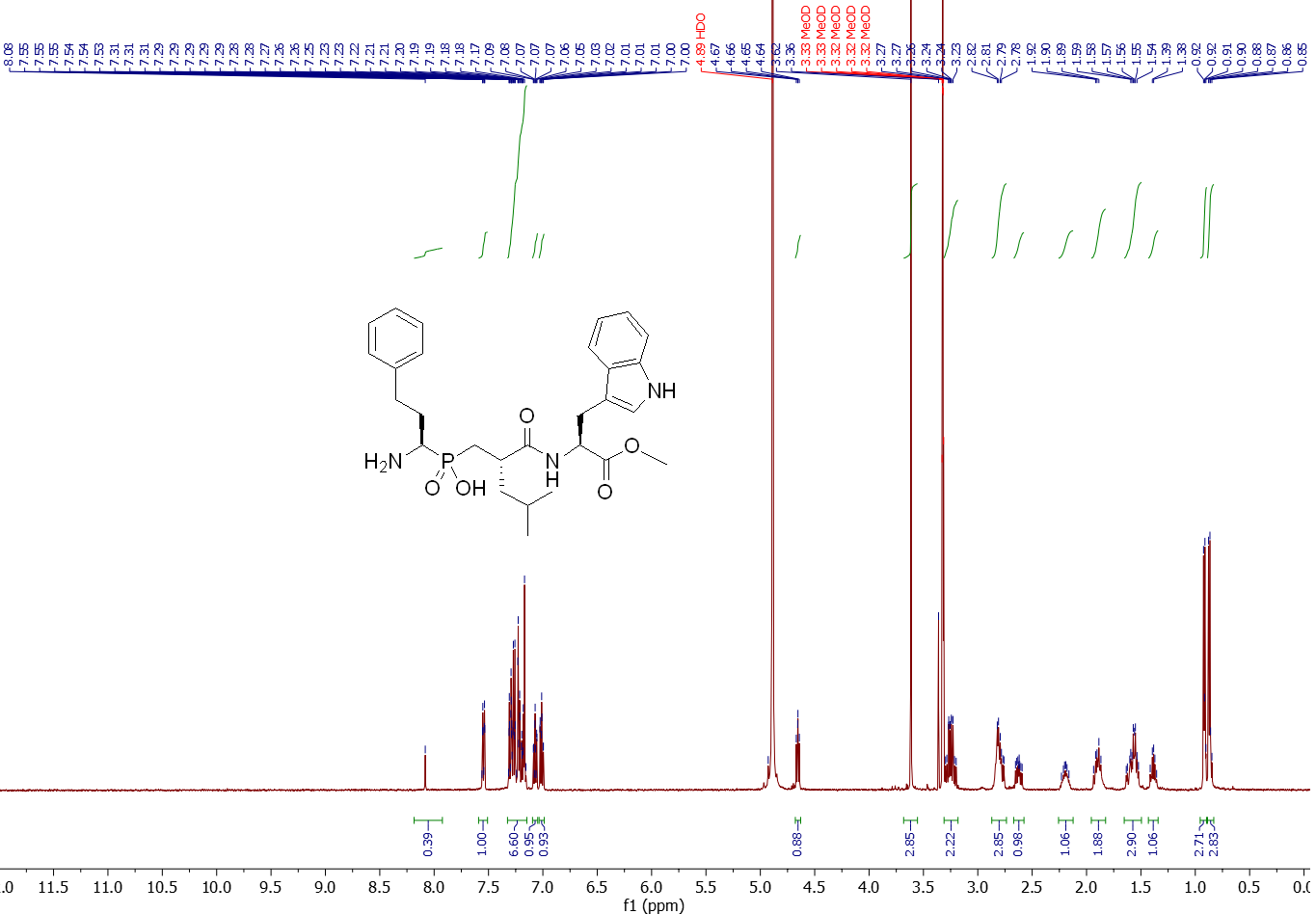


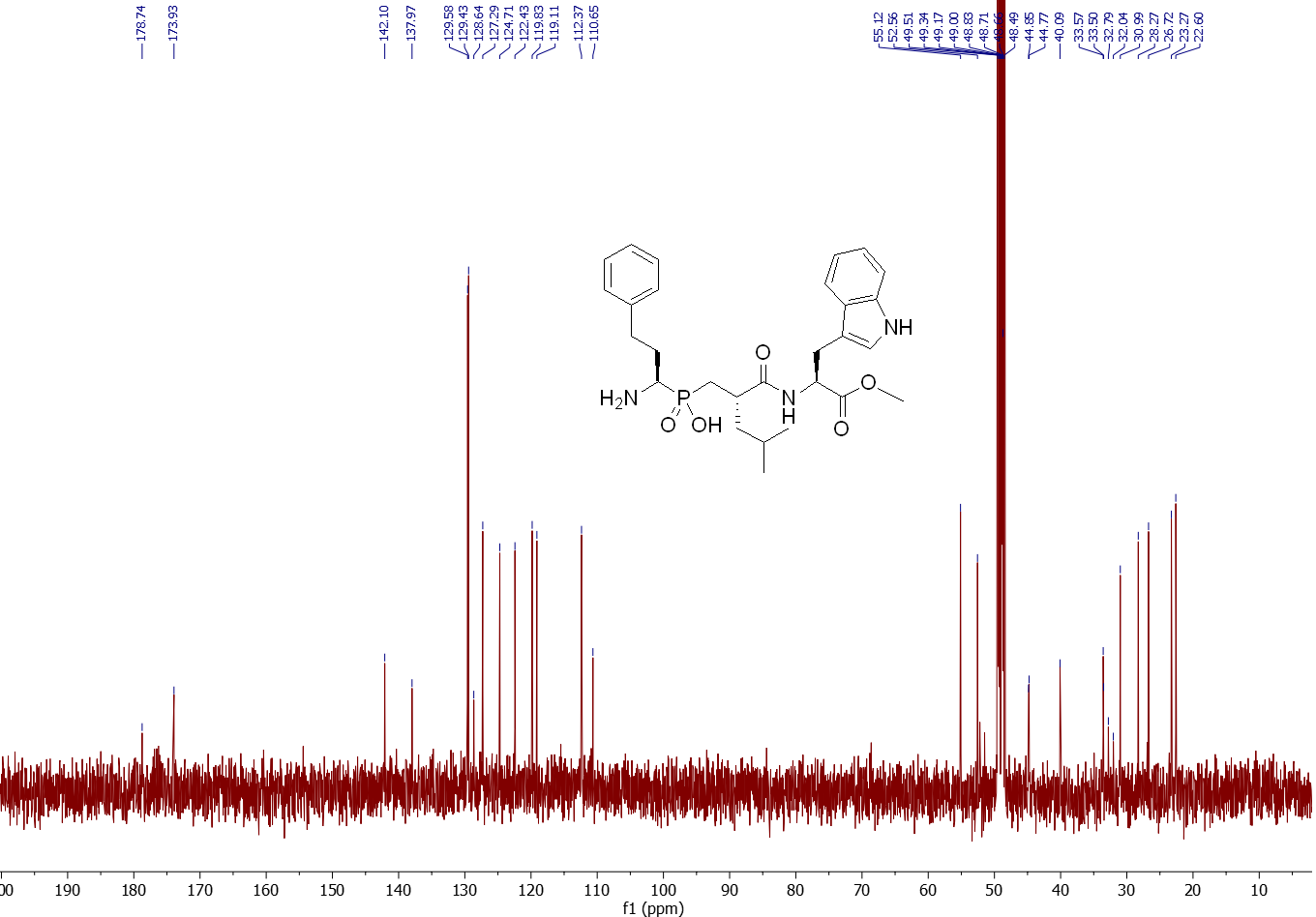


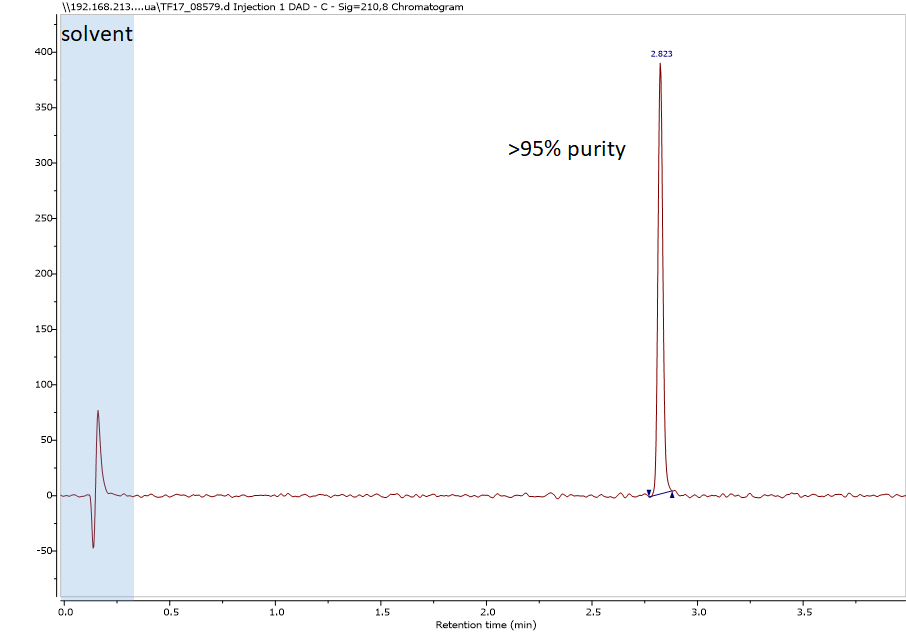

NMR spectra **18**: ^1^H NMR (500 MHz, Methanol-*d*_4_), ^13^C NMR (126 MHz, Methanol-*d*_4_), ^31^P NMR (202 MHz, Methanol-*d*_4_) and LC trace


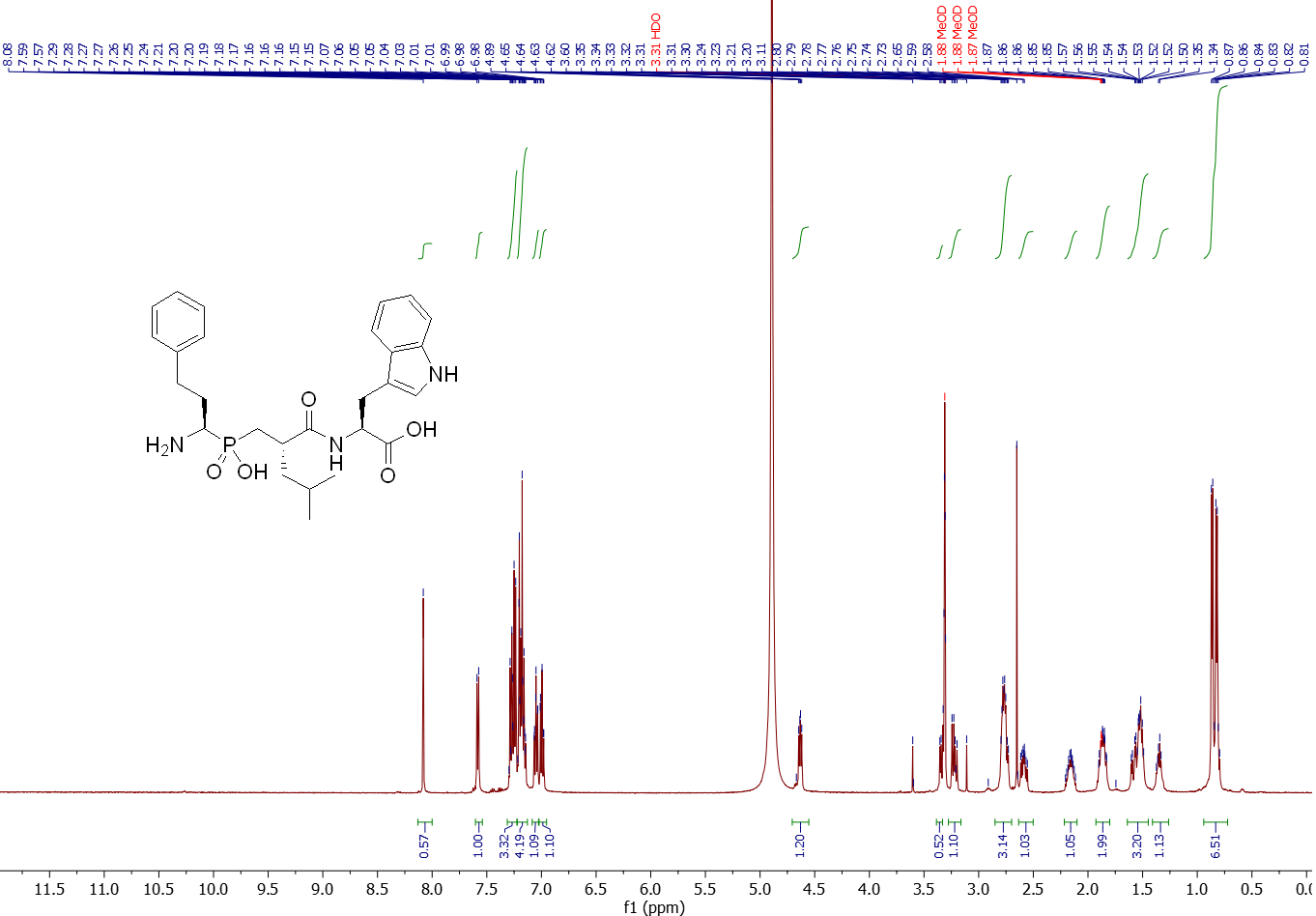


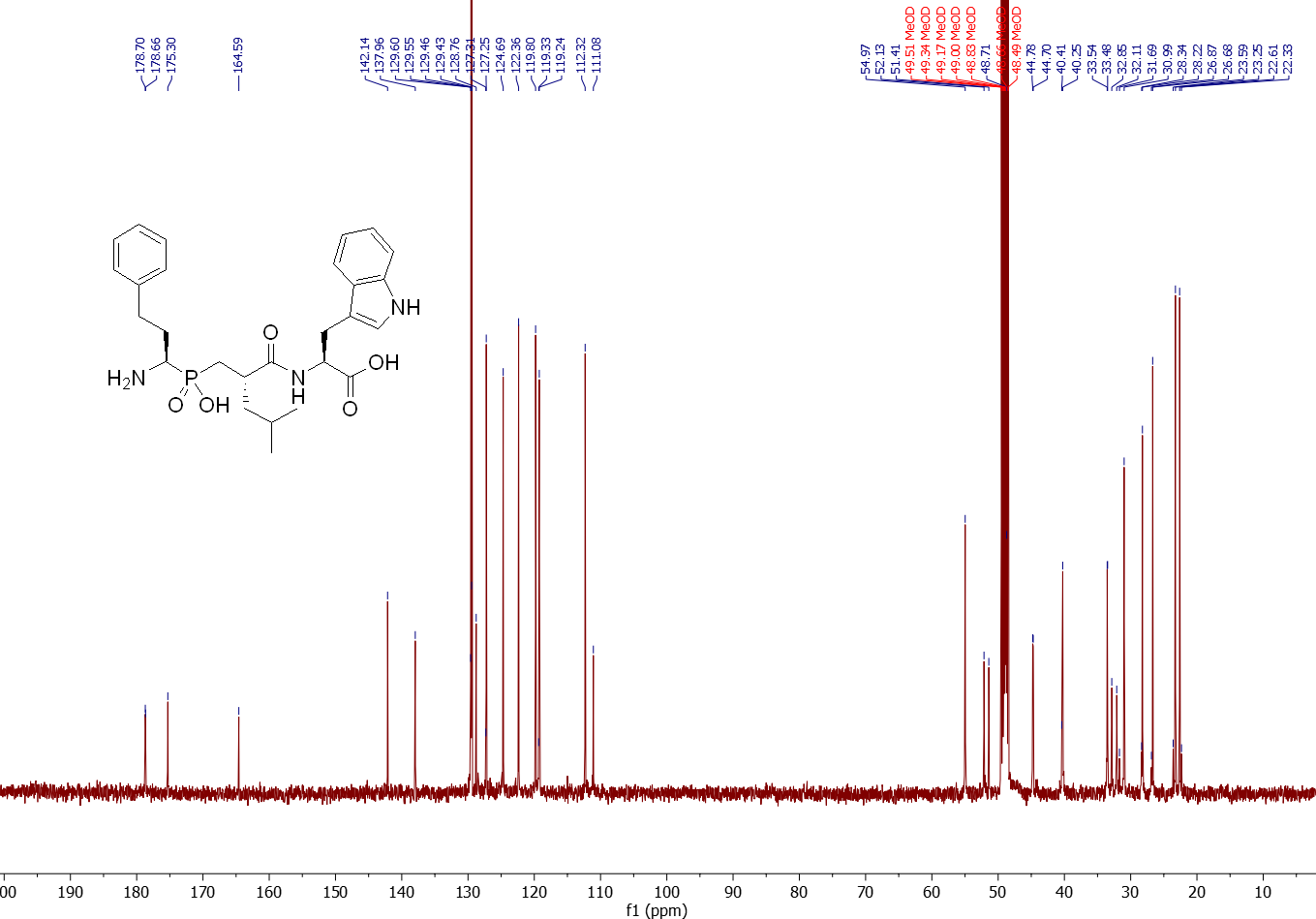


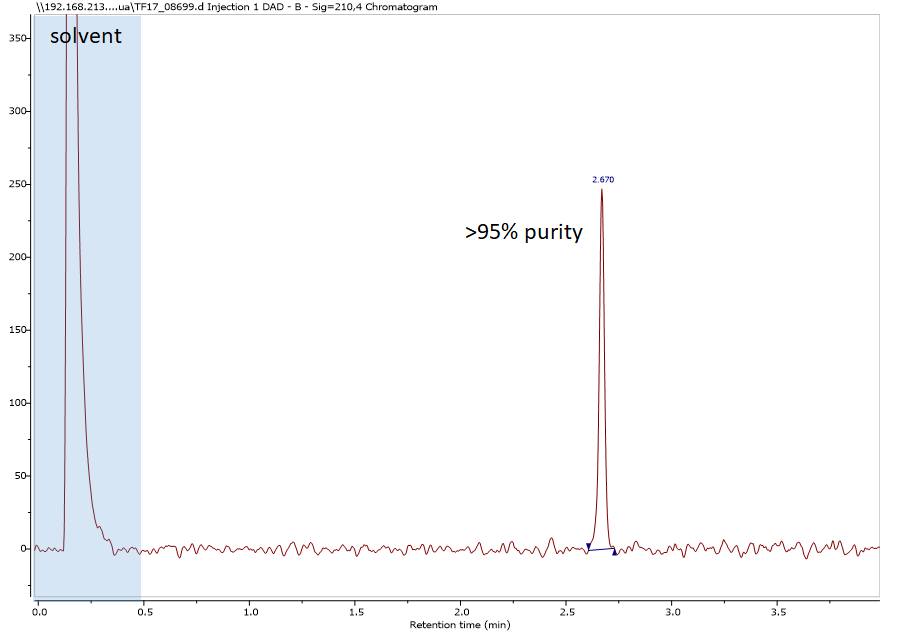

NMR spectra **19**: ^1^H NMR (500 MHz, Methanol-*d*_4_), ^13^C NMR (126 MHz, Methanol-*d*_4_), ^31^P NMR (202 MHz, Methanol-*d*_4_) and LC trace


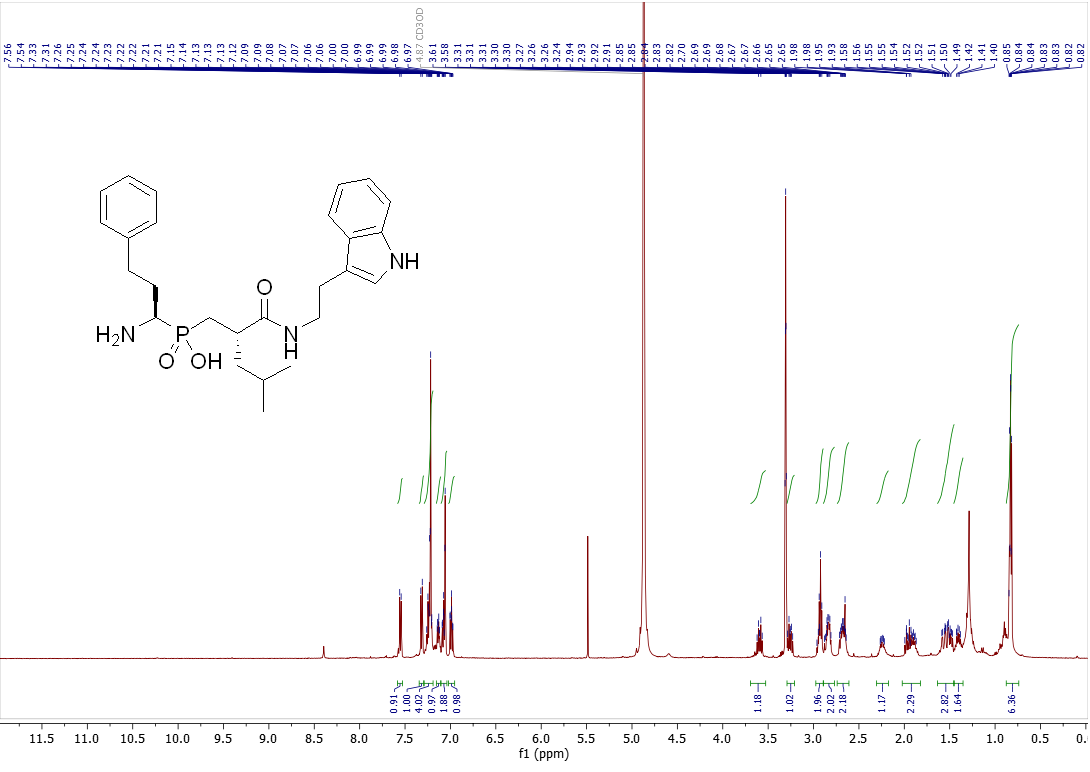


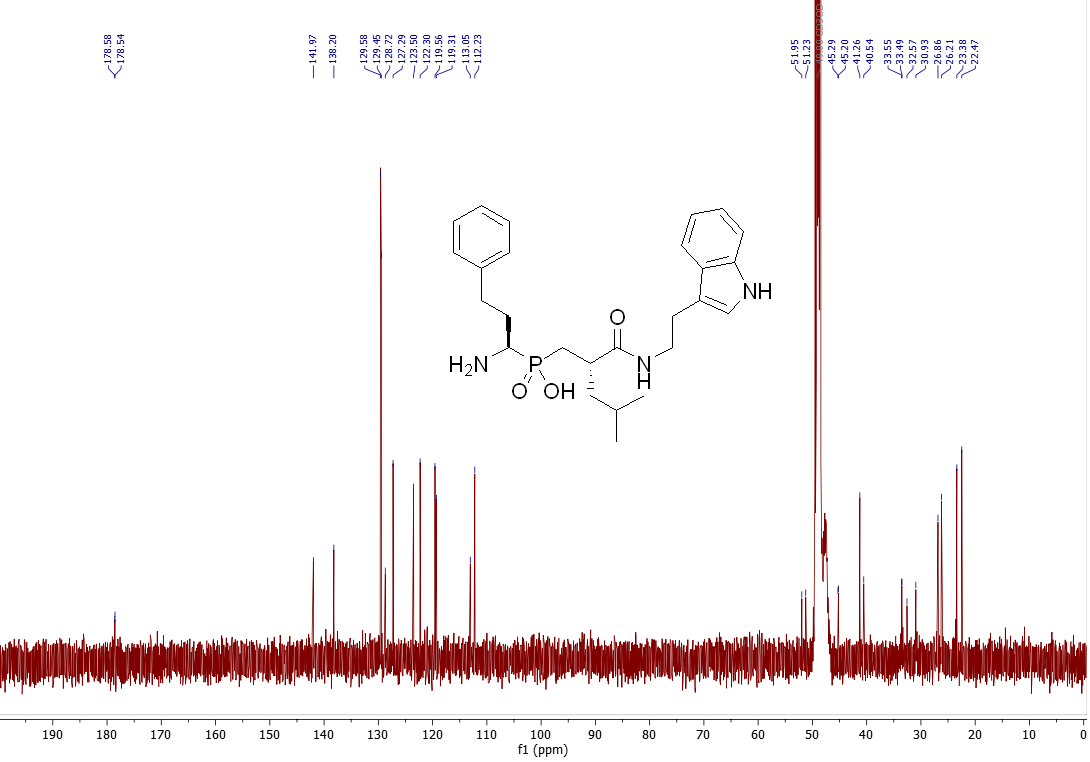


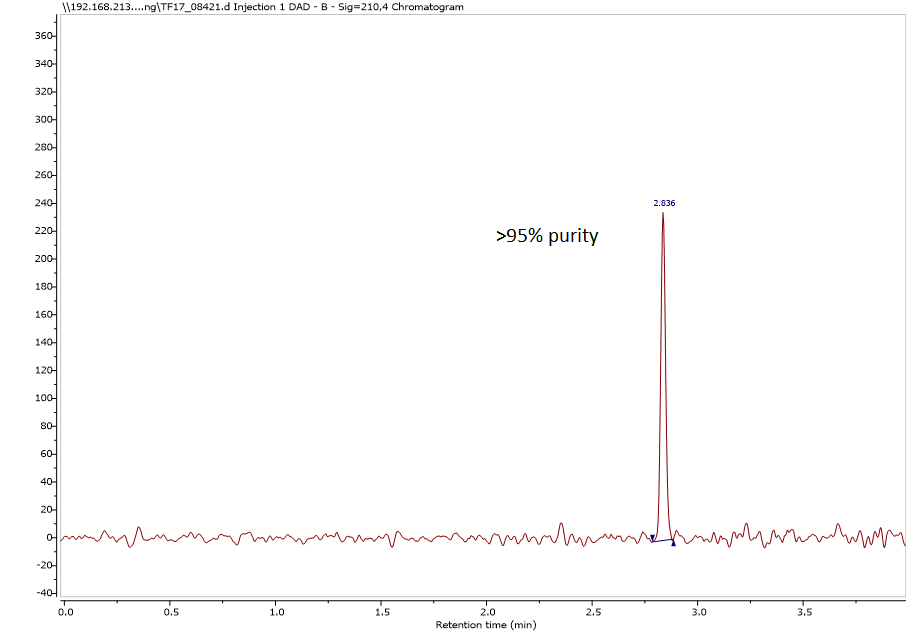

**M1-Aminopeptidase Crystal Structures**

PDB: 6M8P ERAP1/DG013A **1**


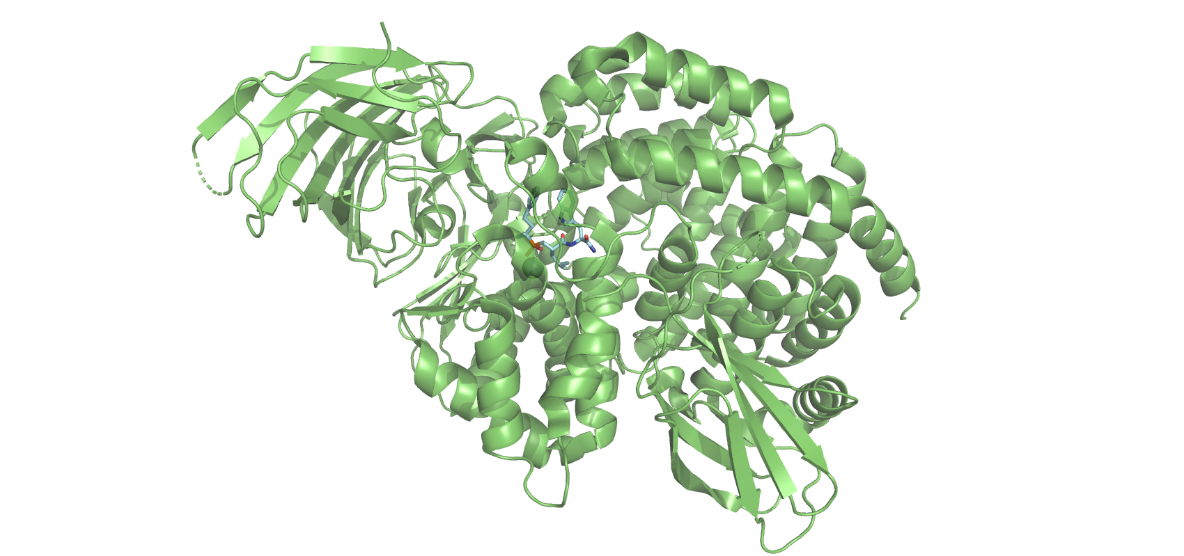

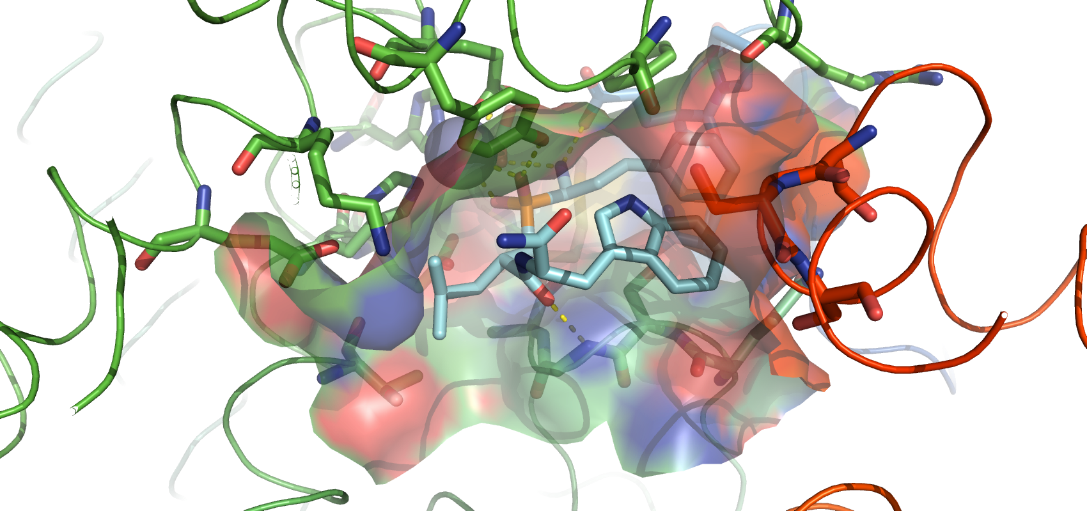


PDB: 4JPS ERAP2/DG013A **1**


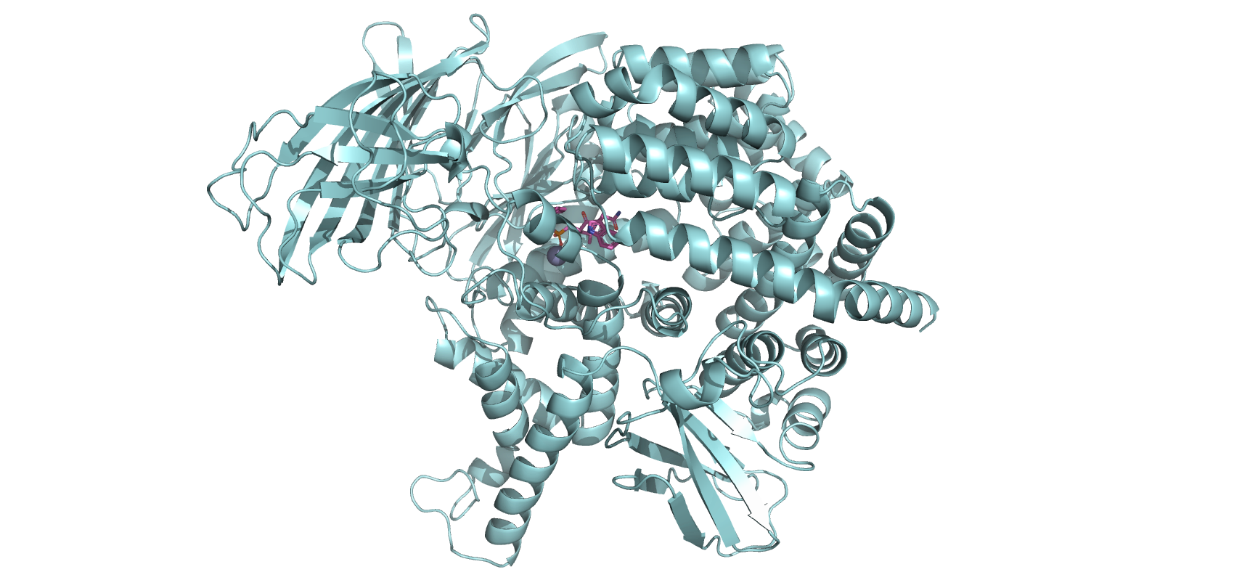

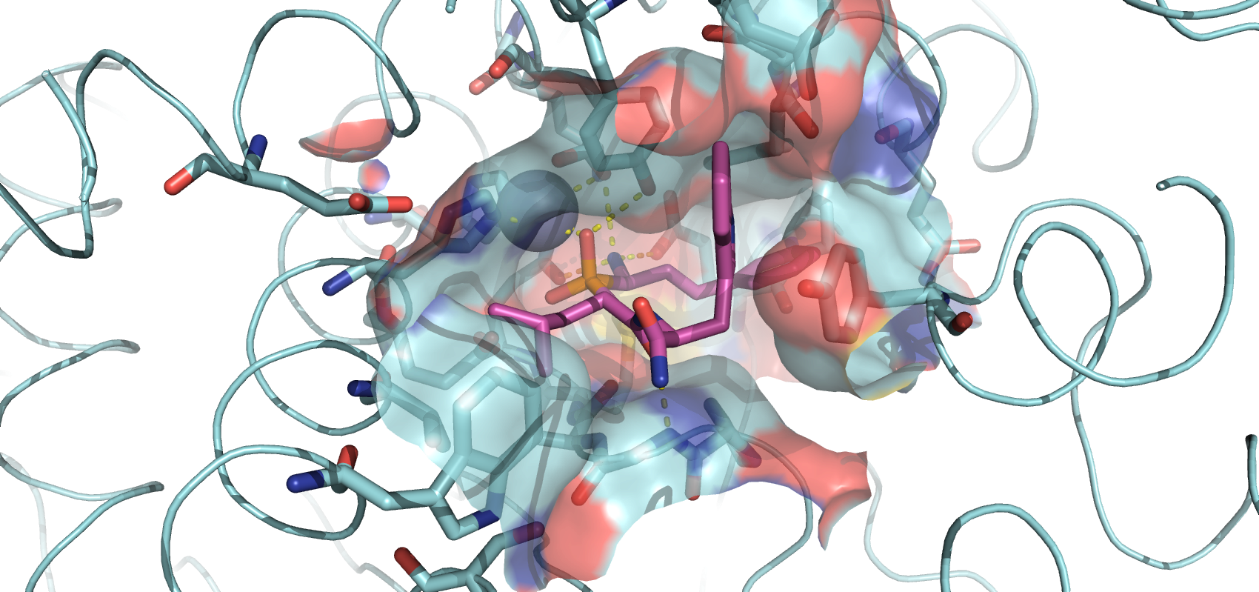


PDB: 4FYR APN/Bestatin


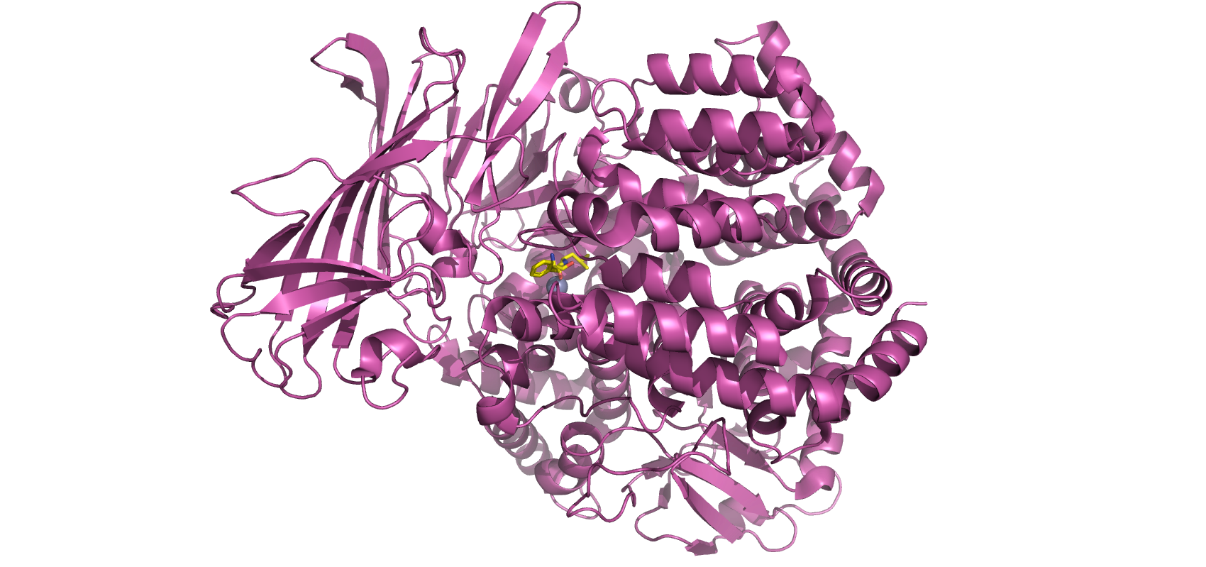

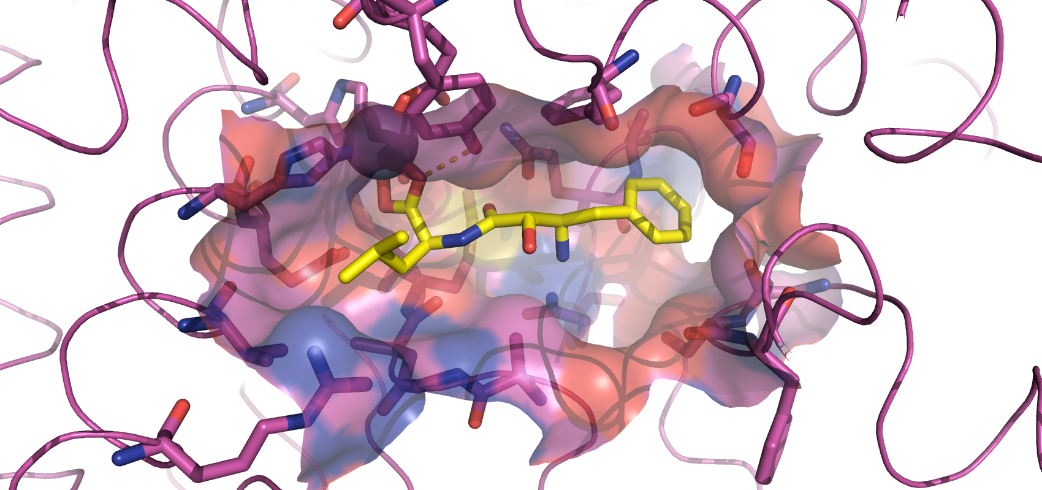


ERAP1/ERAP2/APN Overlay


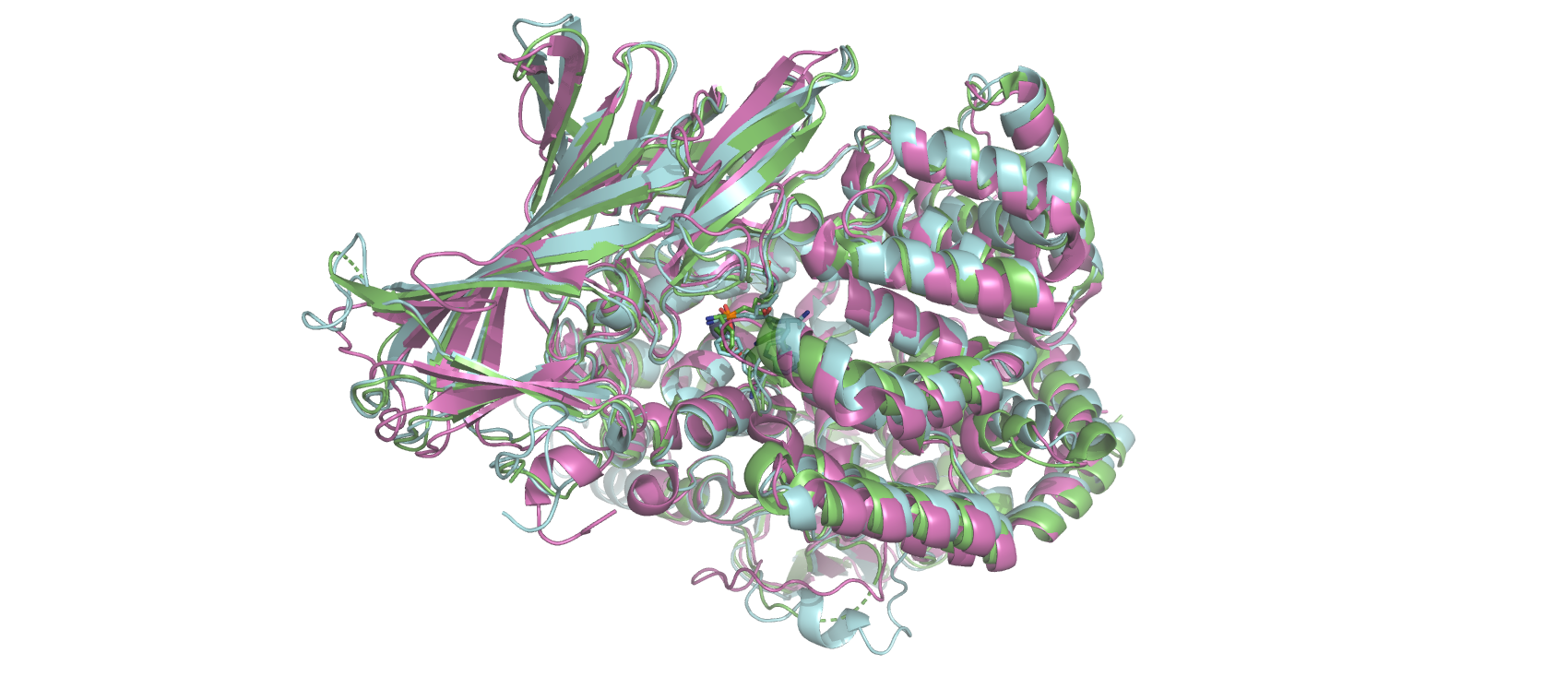

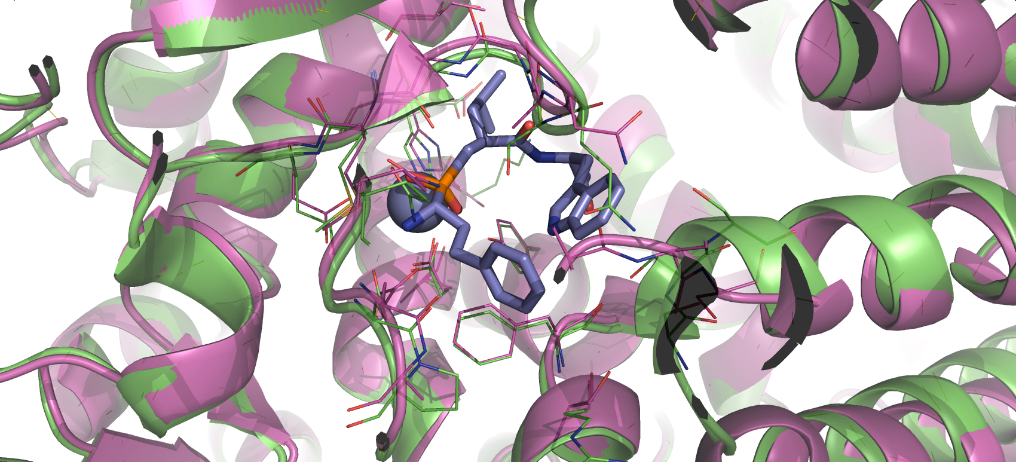


**Biochemical Experimental**

**ERAP1 Protein Synthesis**

Full length human ERAP1 was cloned into the pFastBac1 vector with a tobacco etch virus (rTEV) protease cleavable C-terminal deca-histidine tag followed by a FLAG tag. The generation of recombinant bacmids and baculoviruses, insect cell culture using Hi5 cells, and infections were performed using standard protocols. The supernatant was harvested 120 h post infection and processed using tangential flow filtration (TFF). The buffer composition of the supernatant was adjusted to a final concentration of 50 mM HEPES pH 8.0 and 300 mM NaCl. The supernatant was then loaded on to a 5 ml HisTrap excel column (GE Healthcare) pre-equilibrated in 50 mM HEPES pH 8.0, 500 mM NaCl, 10 mM Imidazole and 10% glycerol. The protein was eluted with an elution buffer containing 50 mM HEPES pH 8.0, 500 mM NaCl, 250 mM Imidazole and 10% glycerol. The eluate was then treated with rTEV protease and dialyzed overnight against 3 L of dialysis buffer (50 mM HEPES pH 8.0, 250 mM NaCl and 10% glycerol). This rTEV cleaved sample was then loaded on to a 5 mL HisTrap excel column (GE Healthcare) pre-equilibrated in 50 mM HEPES pH 8.0, 500 mM NaCl, 10 mM Imidazole and 10% glycerol. The rTEV cleaved sample was recovered in the flow-through of this column. Full-length ERAP1 was then concentrated to 5 mL and applied to a Superdex 200 16/60 (GE Healthcare) gel-filtration column equilibrated in 10 mM HEPES pH 7.0, 100 mM NaCl and 5% glycerol as a final polishing step.

**Sequence:**

MVFLPLKWSLATMSFLLSSLLALLTVSTPSWCQSTEASPKRSDGTPFPWNKIRLPEYVIPVHYDLLIHANLTTLTFWGTTKVEITASQPTSTIILHSHHLQISRATLRKGAGERLSEEPLQVLEHPRQEQIALLAPEPLLVGLPYTVVIHYAGNLSETFHGFYKSTYRTKEGELRILASTQFEPTAARMAFPCFDEPAFKASFSIKIRREPRHLAISNMPLVKSVTVAEGLIEDHFDVTVKMSTYLVAFIISDFESVSKITKSGVKVSVYAVPDKINQADYALDAAVTLLEFYEDYFSIPYPLPKQDLAAIPDFQSGAMENWGLTTYRESALLFDAEKSSASSKLGITMTVAHELAHQWFGNLVTMEWWNDLWLNEGFAKFMEFVSVSVTHPELKVGDYFFGKCFDAMEVDALNSSHPVSTPVENPAQIREMFDDVSYDKGACILNMLREYLSADAFKSGIVQYLQKHSYKNTKNEDLWDSMASICPTDGVKGMDGFCSRSQHSSSSSHWHQEGVDVKTMMNTWTLQKGFPLITITVRGRNVHMKQEHYMKGSDGAPDTGYLWHVPLTFITSKSDMVHRFLLKTKTDVLILPEEVEWIKFNVGMNGYYIVHYEDDGWDSLTGLLKGTHTAVSSNDRASLINNAFQLVSIGKLSIEKALDLSLYLKHETEIMPVFQGLNELIPMYKLMEKRDMNEVETQFKAFLIRLLRDLIDKQTWTDEGSVSERMLRSQLLLLACVHNYQPCVQRAEGYFRKWKESNGNLSLPVDVTLAVFAVGAQSTEGWDFLYSKYQFSLSSTEKSQIEFALCRTQNKEKLQWLLDESFKGDKIKTQEFPQILTLIGRNPVGYPLAWQFLRKNWNKLVQKFELGSSSIAHMVMGTTNQFSTRTRLEEVKGFFSSLKENGSQLRCVQQTIETIEENIGWMDKNFDKIRVWLQSEKLERMAAAENLYFQ

**ERAP2 Protein Synthesis**

Full length human ERAP2 was cloned into the pFastBac1 vector with a C-terminal hexa-histidine tag. The generation of recombinant bacmids and baculoviruses, insect cell culture using Hi5 cells, and infections were performed using standard protocols. The supernatant was harvested 120 h post infection and processed using tangential flow filtration (TFF). The buffer composition of the supernatant was adjusted to a final concentration of 50 mM HEPES pH 8.0 and 300 mM NaCl. The supernatant was then loaded on to a 5 mL HisTrap excel column (GE Healthcare) pre-equilibrated in 50 mM HEPES pH 8.0, 500 mM NaCl, 10 mM Imidazole and 10% glycerol. The protein was eluted with an elution buffer containing 50 mM HEPES pH 8.0, 500 mM NaCl, 250 mM Imidazole and 10% glycerol. Full-length ERAP2 was then concentrated to 5 mL and applied to a Superdex 200 16/60 (GE Healthcare) gel-filtration column equilibrated in 10 mM HEPES pH 7.0, 100 mM NaCl and 5% glycerol as a final polishing step.

**Sequence:**

MFHSSAMVNSHRKPMFNIHRGFYCLTAILPQICICSQFSVPSSYHFTEDPGAFPVATNGERFPWQELRLPSVVIPLHYDLFVHPNLTSLDFVASEKIEVLVSNATQFIILHSKDLEITNATLQSEEDSRYMKPGKELKVLSYPAHEQIALLVPEKLTPHLKYYVAMDFQAKLGDGFEGFYKSTYRTLGGETRILAVTDFEPTQARMAFPCFDEPLFKANFSIKIRRESRHIALSNMPKVKTIELEGGLLEDHFETTVKMSTYLVAYIVCDFHSLSGFTSSGVKVSIYASPDKRNQTHYALQASLKLLDFYEKYFDIYYPLSKLDLIAIPDFAPGAMENWGLITYRETSLLFDPKTSSASDKLWVTRVIAHELAHQWFGNLVTMEWWNDIWLKEGFAKYMELIAVNATYPELQFDDYFLNVCFEVITKDSLNSSRPISKPAETPTQIQEMFDEVSYNKGACILNMLKDFLGEEKFQKGIIQYLKKFSYRNAKNDDLWSSLSNSCLESDFTSGGVCHSDPKMTSNMLAFLGENAEVKEMMTTWTLQKGIPLLVVKQDGCSLRLQQERFLQGVFQEDPEWRALQERYLWHIPLTYSTSSSNVIHRHILKSKTDTLDLPEKTSWVKFNVDSNGYYIVHYEGHGWDQLITQLNQNHTLLRPKDRVGLIHDVFQLVGAGRLTLDKALDMTYYLQHETSSPALLEGLSYLESFYHMMDRRNISDISENLKRYLLQYFKPVIDRQSWSDKGSVWDRMLRSALLKLACDLNHAPCIQKAAELFSQWMESSGKLNIPTDVLKIVYSVGAQTTAGWNYLLEQYELSMSSAEQNKILYALSTSKHQEKLLKLIELGMEGKVIKTQNLAALLHAIARRPKGQQLAWDFVRENWTHLLKKFDLGSYDIRMIISGTTAHFSSKDKLQEVKLFFESLEAQGSHLDIFQTVLETITKNIKWLEKNLPTLRTWLMVNTRHHHHHH

**Aminopeptidase X-AMC Assay**

Aminopeptidase activity was measured according to a protocol adapted from Gandhi et al, Sci. Reports 2011. Briefly, the assay consists in measuring the fluorescence of 7-amido-4-methylcoumarin (AMC) released by the enzymatic hydrolysis of one of the short peptide substrates: Leucine-AMC (Sigma, catalogue # L2145) by the ERAP1 enzyme, Arginine-AMC (Sigma, catalogue # A2027) by the ERAP2 enzyme or Alanine-AMC (Sigma, catalogue #A4302) by the APN (ANPEP/CD13) enzyme. APN enzyme was purchased from Sino Biologics via STRATECH Scientific, UK (catalogue#10051-H08H), while the ERAP enzymes were produced in house. Phosphinic acid compounds were added to 384 well ProxiPlate FX (Perkin Elmer) on Echo550 acoustic dispenser (Labcyte Inc., Beckman-Coulter) at a final concentration varying between 1 nM and 220 µM, in 2.1% DMSO. For ERAP activity measurements, assays were performed in 10 µL of 50 mM Tris/HCl, pH8.0, 0.1 M NaCl, containing either 250 µM L-AMC substrate with 5 nM ERAP1 enzyme, or 10 µM R-AMC substrate with 10 nM ERAP2 enzyme. For APN activity measurement, assay was performed in 10µL of 50 mM Tris/HCl, pH7.4, containing 100 µM Ala-AMC substrate and 0.96 nM enzyme. After 30 minutes of reaction at room temperature, the fluorescence was red on a Pherastar FSX (BMG LabTech) or an Envision (Perkin Elmer) plate readers, with excitation and emission filters at 350 nm and 460 nm, respectively.

**Example Data and analysis for DG013A 1**

**Permeability Assay**

Caco-2 Assay Protocol

P_app_ (apparent permeability) was determined in the Caco-2 human colon carcinoma cell line (ATCC). Cells were maintained (DMEM with 10% fetal bovine serum, penicillin, and streptomycin) in a humidified atmosphere with 5% CO_2_/95% air for 10 days. Cells were plated out onto a cell culture assembly plate (Millipore, UK), and monolayer confluency was checked using a TEER electrode prior to the assay. Media was washed off and replaced in the appropriate apical and basal wells with HBSS buffer (pH 7.4) containing compound (10 μM, 1% DMSO). The Caco-2 plate was incubated for 2 h at 37 °C, and Lucifer Yellow was used to confirm membrane integrity after the assay. Samples from the apical and basolateral chambers were analyzed using a Waters TQ-S LC-MS/MS system.

| Entry | Compd | Structure | P_app_ A:B  (x10^-6^ cm/s) | P_app_ B:A  (x10^-6^ cm/s) | Efflux Ratio | Recovery A:B  (%) | Recovery B:A  (%) |
| --- | --- | --- | --- | --- | --- | --- | --- |
| 1 | DG013A **1** |  | <1.0 | <1.0 | NA | 97 | 100 |
| 2 | DG013B **2** |  | <1.0 | <1.0 | NA | 83 | 90 |
| 3 | **3** |  | <1.0 | 4.3 | >12 | 79 | 84 |
| 4 | **14** |  | <1.0 | <1.0 | Na | 2 (Ester hydrolysis observed) | 84 |
| 5 | **15** |  | <1.0 | <1.0 | NA | 83 | 96 |
| 6 | **16** |  | 1.5 | 12 | 8.0 | 100 | 100 |

**HCT116 Proliferation Assay**

Origin of the human colon carcinoma cell lines used in this study: The HCT116 cell line was obtained from the ICR Cell Bank. It was passaged in vivo in athymic mice to increase its tumorigenicity and reliability as a xenograft, and used within 10 passages of banked stocks. Prior to use, the cells were analyzed by short tandem repeat (STR) profiling. Polymorphic STR loci were amplified using a polymerase chain reaction (PCR) primer set. The PCR product (each locus being labelled with a different fluorophore) was analyzed simultaneously with size standards using automated fluorescent detection. The number of repeats at 10 different loci (as recommended by the American Type Culture Collection, ATCC) was used to define the STR profile and this was cross-referenced with online databases to confirm authenticity. Using this method, the in vivo subline showed an acceptable 85.71 % identity with the ATCC reference line (LGC Promochem, UK). The cells were free of mycoplasma contamination as determined by a sensitive nested PCR protocol (Venor GeM kit, Minerva Biolabs, Germany). Cells were grown in DMEM/10% FBS, 1% pyruvate in 5% CO_2_.

The CellTiter-Glo 2.0 viability (Promega) assay provides a homogenous, luminescence method for estimating the number of viable cells. It quantifies the amount of ATP present, to measure the metabolic capacity of cells which is an indicator of cell viability. Viable cells are able to contribute Mg2+, ATP, which is able to catalyze the mono-oxygenation of luciferin by luciferase. Briefly, cells (2 x 10^3^ cells/mL) were seeded into 384-well plates and compounds (at a range of concentrations) were added using the ECHO 650 liquid handler (Labcyte, USA) and then left at 37 ^o^C for 48 or 72 hours. CellTiter-Glo 2.0 reagent was added to each well and left at 37 ^o^C for 1 h. Luminescence was measured using the Envision machine (Perkin Elmer, UK). The 50% growth inhibitory concentration (GI_50_) was determined by fitting the data to a dose-response curve without limits using non-linear regression. Each concentration was tested twice.

Day1. DG013A was transferred into each well of a 384-well plate (Greiner - 781091) at a range of concentrations (0.075 to 30 µM) to construct an 8-point dose response curve (final DMSO concentration 0.3%) using the Echo 650T Liquid Handler (Labcyte). HCT116 cells were then seeded at a density of 2x10^3^ cells per well in 25 uL DMEM media (+ 1% pyruvate and 10% FBS) and were incubated in compound for 48 or 72 hours at 37^O^C.

Day4. Added 25 uL of CellTiter-Glo 2.0 (Promega) reagent to each well as per manufacturer’s instructions to the 72hr treatment plate.

Figure. Anti-proliferative effect of DG013A on HCT116 cells

Calculated Physicochemical Properties

| Entry | Compd | Structure | tPSA | cLogP |
| --- | --- | --- | --- | --- |
| 1 | DG013A **1** |  | 148 | 3.2 |
| 2 | DG013B **2** |  | 148 | 3.2 |
| 3 | **3** |  | 160 | 5.4 |
| 4 | **14** |  | 131 | 4.3 |
| 5 | **15** |  | 142 | 4.2 |
| 6 | **16** |  | 104 | 4.4 |

Properties calculated using Chemdraw 19.1.0.8

1. Lämmerhofer, M.; Hebenstreit, D.; Gavioli, E.; Lindner, W.; Mucha, A.; Kafarski, P.; Wieczorek, P. High-performance liquid chromatographic enantiomer separation and determination of absolute configurations of phosphinic acid analogues of dipeptides and their α-aminophosphinic acid precursors. *Tetrahedron: Asymmetry* **2003**, *14*, 2557-2565. [↑](#endnote-ref-1)
2. Baylis, E. K.; Campbell, C. D.; Dingwall, J. G. 1-Aminoalkylphosphonous acids. Part 1. Isosteres of the protein amino acids. *J. Chem. Soc., Perkin Trans. 1 (1972-1999)* **1984**, 2845-2853. [↑](#endnote-ref-2)
3. Kokkala, P.; Mpakali, A.; Mauvais, F.-X.; Papakyriakou, A.; Daskalaki, I.; Petropoulou, I.; Kavvalou, S.; Papathanasopoulou, M.; Agrotis, S.; Fonsou, T.-M.; van Endert, P.; Stratikos, E.; Georgiadis, D. Optimization and structure–activity relationships of phosphinic pseudotripeptide inhibitors of aminopeptidases that generate antigenic peptides. *J. Med. Chem.* **2016**, *59*, 9107-9123. [↑](#endnote-ref-3)
4. # Yao, Q.; Yuan, C. Enantioselective Synthesis of *H*-Phosphinic Acids Bearing Natural Amino Acid Residues. *J. Org. Chem.* **2013**, *78*, 6962-6974.

   [↑](#endnote-ref-4)
5. Chen, H.; Noble, F.; Mothé, A.; Meudal, H.; Coric, P.; Danascimento, S.; Roques, B. P.; George, P.; Fournié-Zaluski, M.-C. Phosphinic derivatives as new dual enkephalin-degrading enzyme inhibitors: Synthesis, biological properties, and Phosphinic derivatives as new dual enkephalin-degrading enzyme inhibitors: Synthesis, biological properties, and anticeptive activities. *J. Med. Chem.* **2000**, *43*, 1398-1408. [↑](#endnote-ref-5)
6. Kokkala, P.; Mpakali, A.; Mauvais, F.-X.; Papakyriakou, A.; Daskalaki, I.; Petropoulou, I.; Kavvalou, S.; Papathanasopoulou, M.; Agrotis, S.; Fonsou, T.-M.; van Endert, P.; Stratikos, E.; Georgiadis, D. Optimization and structure–activity relationships of phosphinic pseudotripeptide inhibitors of aminopeptidases that generate antigenic peptides. *J. Med. Chem.* **2016**, *59*, 9107-9123. [↑](#endnote-ref-6)
7. Liu, X.; Hu, E.; Tian, X; Mazur, A.; Ebetino, F. H. Enantioselective synthesis of phosphinyl peptidomimetics via an asymmetric Michael reaction of phosphinic acids with acrylate derivatives. *J. Organomet. Chem.* **2002**, *646*, 212-222. [↑](#endnote-ref-7)
8. Evans, D. A.; Black, W. C. Total Synthesis of (+)-A83543A [(+)-Lepicidin A]. *J. Am. Chem. Soc.* **1993**, *115*, 4497-4513. [↑](#endnote-ref-8)
